# A combined fMRI and EMG study of emotional contagion following partial sleep deprivation in young and older humans

**DOI:** 10.1038/s41598-020-74489-9

**Published:** 2020-10-21

**Authors:** Sandra Tamm, Johanna Schwarz, Hanna Thuné, Göran Kecklund, Predrag Petrovic, Torbjörn Åkerstedt, Håkan Fischer, Mats Lekander, Gustav Nilsonne

**Affiliations:** 1 Stress Research Institute, Department of Psychology, Stockholm University, Stockholm, Sweden; 2 Department of Clinical Neuroscience, Karolinska Institutet, Stockholm, Sweden; 3 Department of Psychology, University of Glasgow, Glasgow, UK; 4 Department of Psychology, Stockholm University, Stockholm, Sweden

**Keywords:** Sleep, Sleep deprivation, Amygdala, Human behaviour, Circadian rhythms and sleep, Emotion

## Abstract

Sleep deprivation is proposed to inhibit top-down-control in emotion processing, but it is unclear whether sleep deprivation affects emotional mimicry and contagion. Here, we aimed to investigate effects of partial sleep deprivation on emotional contagion and mimicry in young and older humans. Participants underwent partial sleep deprivation (3 h sleep opportunity at the end of night), crossed-over with a full sleep condition in a balanced order, followed by a functional magnetic resonance imaging and electromyography (EMG) experiment with viewing of emotional and neutral faces and ratings of emotional responses. The final sample for main analyses was *n* = 69 (*n* = 36 aged 20–30 years, *n* = 33 aged 65–75 years). Partial sleep deprivation caused decreased activation in fusiform gyri for angry faces and decreased ratings of happiness for all stimuli, but no significant effect on the amygdala. Older participants reported more anger compared to younger participants, but no age differences were seen in brain responses to emotional faces or sensitivity to partial sleep deprivation. No effect of the sleep manipulation was seen on EMG. In conclusion, emotional contagion, but not mimicry, was affected by sleep deprivation. Our results are consistent with the previously reported increased negativity bias after insufficient sleep.

The Stockholm sleepy brain study: effects of sleep deprivation on cognitive and emotional processing in young and old. https://clinicaltrials.gov/ct2/show/NCT02000076.

## Introduction

Sleep problems are widespread, affecting about one third of the general population, of which about one in three reports serious problems^1,2^. Epidemiological studies have suggested that poor sleep is prospectively connected to psychiatric morbidity, especially depression^3^, where emotional dysfunction forms part of the symptomatology. Acute experimental sleep deprivation has been associated with decreased social emotional functions, such as slower voluntary facial responsiveness to emotional stimuli^4^, less accuracy in emotional face recognition^5,6^, and an increased negativity bias^7^. No study so far has investigated whether any type of sleep restriction affects emotional contagion and mimicry.

Facial emotional signals play a vital part in human social interplay^8,9^. Lipps suggested that observation of others’ emotional expressions causes mimicry as well as a merging of emotions^10,11^ and emotional contagion was defined by Hatfield et al.^12^ as ‘the tendency to automatically mimic and synchronize expressions, vocalizations, postures, and movements with those of another person and, consequently, to converge emotionally’. In other words, emotional mimicry, which can be studied by recording muscle activity from facial muscles involved in, for example, smiling^13^, constitutes a part of emotional contagion. The facial feedback hypothesis states that the facial muscles function as a feedback system for a person’s own experience of emotion^14,15^ and it has been suggested that similar feedback signals are involved in perception of others’ emotion^16^. Emotional mimicry develops early in infanthood^17^, and even though the empirical base is limited, emotional mimicry has been suggested to constitute one form of empathy^15,18^. To understand mechanisms behind the effect of sleep deprivation on complex social emotional functions, it is necessary to investigate both less complex low-level functions, such as emotional mimicry and contagion, and more complex functions such as empathy and emotional regulation. The Stockholm Sleepy Brain study comprised three task fMRI experiments to investigate effects of partial sleep deprivation on emotional mimicry and contagion, empathy, and emotional regulation. Results from the experiments on empathy and emotional regulation have been previously reported^19,20^. Here we focus on the effect of partial sleep deprivation on emotional mimicry and contagion.

Perception and processing of both emotional and non-emotional faces have been studied repeatedly using brain imaging^21,22^. Such studies have reliably demonstrated activation during face processing in a set of areas in the occipital and temporal cortex^23^, where the inferior occipital gyrus is related to initial perception of the face, the superior temporal sulcus is proposed to process changeable aspects of the face (lip movement, gaze direction), and the lateral fusiform area is proposed to be involved in the representation of identity^23^. Processing of emotional faces involves additional areas, most importantly the amygdala, reasonably consistently activated for faces expressing fear, anxiety and sadness, but less consistently for happiness^24–26^. Therefore, if sleep deprivation affects emotional mimicry, a possible mechanism would be through altered amygdala reactivity, as suggested for other types of emotional processing^27,28^.

In recent years, links between sleep and emotional processing have been studied from several angles, using both behavioural outcomes and neuroimaging. A much-cited neuroimaging finding is that insufficient sleep is associated with increased amygdala reactivity^27–30^. This was initially shown with experimental sleep deprivation for negative stimuli^27^ and later for fearful faces^31^ and a proposed mechanism was decreased connectivity between prefrontal cortex and amygdala^27^. In behavioural studies, sleep deprivation has been shown to cause a negativity bias in a number of studies^7,32,33^, i.e., a shift towards more negative and less positive emotion. There are however other results suggesting different interpretations, for example, showing that sleep deprived subjects had lower intensity ratings of both happy and angry faces^6^ and similarly, patients with insomnia have been shown to rate lower intensity of fear and sadness expressions in a face task^34^. The latter results would suggest a blunting of responses rather than a shift towards negativity. Emotional contagion and mimicry, as defined above, have not been investigated after experimentally manipulating sleep. However, Brand and colleagues suggested that poor sleep as subjectively experienced among adolescents is associated with specific impairments in emotional competence and empathy (as measured with questionnaires)^35^. Moreover, Tempesta and colleagues showed that sleep deprivation was associated with lower scores on the affective and cognitive subscales of Basic Empathy Scale, while no differences were observed for implicit or explicit emotional empathy during the Multifaced Empathy Test^36^. While implicit emotional empathy in that study is relatively similar to emotional contagion, facial mimicry was not recorded.

Not only responses to others’ faces have been studied after sleep deprivation, but also effects of sleep deprivation on the sleep deprived’s facial expression. In general, sleep deprivation is associated with being perceived as less healthy and attractive^37^. Specifically, sleep deprivation has been shown to be associated with fewer facial movements to sad and amusing film clips^38^, and slower facial electromyographic (EMG) responses to positive and negative scenes, as well as to happy and angry faces^4^. In summary, there is evidence that the perception of emotional faces, as well as facial expressions, might be altered after sleep deprivation. No study has however investigated the effect of *partial* sleep deprivation, an ecologically more valid condition than total sleep deprivation, on perception and responses to emotional faces, and no study has investigated the effect of any sleep manipulation on emotional contagion and mimicry using both functional magnetic resonance imaging (fMRI), ratings and EMG of facial muscles. As indicated above, studying how these low-level emotional functions are affected by short sleep might contribute to the understanding of how social interactions are affected by insufficient sleep, but also potentially contribute to the understanding of how sleep problems can lead to for example depressive symptoms^3^.

Most studies of sleep deprivation and emotional functioning have been performed in young healthy participants. We have shown in several publications that effects of sleep restriction generalize poorly across age groups^19,20,39^. Older and younger participants respond differently not only to emotional stimuli per se, but also to a sleep manipulation^40^. Since aging is associated with less accuracy in recognising emotions^41,42^ and a positivity bias for emotion^43^, as well as entailing decreased sensitivity to sleep deprivation^44^, age differences could also explain some of the inconsistencies in previous data. We here included both younger and older adult participants, to increase generalizability and to explore if aging is associated with changed responses to partial sleep deprivation, for emotional contagion and mimicry. Another factor that might contribute to the inconsistency in previous literature is the use of different types of sleep manipulations, such as partial or total sleep deprivation or correlational analyses based on recordings of habitual sleep. Since partial sleep deprivation might be more similar to common sleep disturbance but still allows experimental control, here we investigated emotional contagion and mimicry after experimentally inducing partial sleep deprivation. Based on the partly inconsistent literature reported above, we expected sleep restriction to be associated with a negativity bias for emotional ratings^7,32^, a less sensitive facial feedback system^4,38^ and increased amygdala reactivity to emotional faces^27,31^.

## Aims

We aimed to investigate the effect of partial sleep deprivation on emotional contagion and facial emotional mimicry. The main hypotheses [a full list of pre-specified hypotheses and an analysis plan can be found at the Open Science Framework (https://osf.io/zuf7t/)] were that: (1) Partial sleep deprivation will cause decreased emotional contagion, measured as ratings of happiness and increased ratings of anger in response to emotional faces, i.e. a negativity bias. (2) Partial sleep deprivation will cause decreased emotional mimicry, measured as activity in *M. corrugator* and in *M. zygomaticus* during exposure to both happy and angry faces, i.e. a less sensitive facial feedback system. (3) Angry and happy faces will cause greater blood oxygen dependent (BOLD) responses in the amygdala and fusiform gyrus than neutral faces and PSD will interact with this effect to cause greater increases to angry faces (in line with the findings of Yoo et al.^27^ and Motomura et al.^31^), whereas the direction of the interaction effect is not specified for happy faces. Additionally, we aimed to explore the difference between younger and older participants for all the main outcomes as well as the interaction between aging and sleep deprivation for emotional contagion and mimicry.

## Methods

### Study design

The present study reports findings from one of the experimental tasks from the Stockholm Sleepy Brain Study 1. The Stockholm Sleepy Brain Study 1 was an experiment on the effects of partial sleep deprivation (3 h of sleep) in younger (20–30 years) and older (65–75 years) healthy participants, in a randomised cross-over design. We have previously published a detailed description of study procedures and the resulting dataset^45^. Other reports based on the same dataset describe resting state connectivity^46^, polysomnography data^40^, empathy^20^, emotional regulation^19^ and morphometry^47^. The study was approved by the Regional Ethics Review board of Stockholm (2012/1565-32 and 2012/1870-32) and all participants gave written informed consent. Experiments were performed in accordance with the Declaration of Helsinki and applicable local regulations. The project was preregistered at clinicaltrials.gov (https://clinicaltrials.gov/ct2/show/NCT02000076), registration number NCT02000076, registered on 03/12/2013.

### Participants

As described in Nilsonne et al.^45^, participants were recruited by poster advertising on campus sites in Stockholm, on the studentkaninen.se website, and through newspaper ads. Putative participants were screened for inclusion/exclusion criteria using an online form and eligibility was confirmed in an interview upon arrival to the scanning site. Criteria for inclusion were, first, those required to undergo fMRI procedures and to use the hand-held response box, namely: no ferromagnetic items in body, not claustrophobic, not pregnant, no refractive error exceeding 5 dioptres, not color-blind, and right-handed. In addition, participants were required to be 20–30 or 65–75 years old (inclusive), to have no current or past psychiatric or neurological illness, including addiction, to not have hypertension or diabetes, to not use psychoactive or immune-modulatory drugs, to not use nicotine every day, and to have a habitual daily caffeine intake corresponding to 4 cups of coffee at most. A further criterion was to not study, have studied, or be occupied in the fields of psychology, behavioural science, or medicine, including nursing and other allied fields. The Insomnia Severity Index (ISI)^48,49^, the depression subscale of the Hospital Anxiety and Depression scale (HADS)^50,51^ and the Karolinska Sleep Questionnaire (KSQ)^52^ were used to exclude participants with insomnia symptoms or depression, irregular sleep patterns, or excessive snoring. The HADS scale assesses symptoms of depression and anxiety and we excluded anyone reporting ≥ 8 on the depression subscale. The ISI measures insomnia symptoms and we excluded participants rating 15 or above. For KSQ, we excluded all participants reporting snoring or sleep apnea symptoms more than 3 times a week. For practical reasons, participants were also required to understand and speak Swedish fluently and to live in the greater Stockholm area. Participants were paid 2500 SEK (approx. 280 Euro/360 USD), subject to tax. They were also offered taxi travel to and from the MRI imaging centre, in order to avoid traffic incidents following sleep deprivation. The full sample consisted of 47 younger and 39 older participants, but because of drop out after enrolment, data loss (including participants where parts of the amygdala had fallen outside the field of view, see below), unsuccessful interventions (described in detail in Refs.^20,45^) and movement in the scanner and failed normalisation (see below) the number of participants included for fMRI analyses of the effect of the intervention was 36 younger and 33 older.

### Sleep measures

As described in Ref.^45^, healthy volunteers underwent MRI scanning on two occasions approximately one month apart after normal sleep and partial sleep deprivation in a counter-balanced and randomized fashion. In the sleep restriction condition, participants were instructed to sleep 3 h in the end of their normal sleep period. As described in Åkerstedt et al.^40^ and in Nilsonne et al.^45,46^ polysomnography (PSG) recording took place in the homes of the participants during both the experimental and the control nights, using a solid state, portable sleep recorder (Embla system for the majority of recordings and Vitaport system for a few). Standard electrode (silver/silver chloride) montage for EEG sleep recording was used (C3, C4 referenced to the contralateral mastoid). Additionally, two sub-mental electrodes were used for electromyography (EMG) and one electrode at each of the outer canthi of the eyes were used for electrooculography (EOG). Sleep staging and respiratory analyses were performed according to the classification criteria of the AASM^53^ and as implemented in the Siesta group computer assisted scoring procedure^54^. For analyses of intervention effects, we only included participants who fulfilled previously reported criteria of having slept < 4 h in the sleep deprivation condition, and a difference between the two conditions > 2 h^45^.

### Experimental paradigm and ratings of happiness and anger

**Figure 1:**
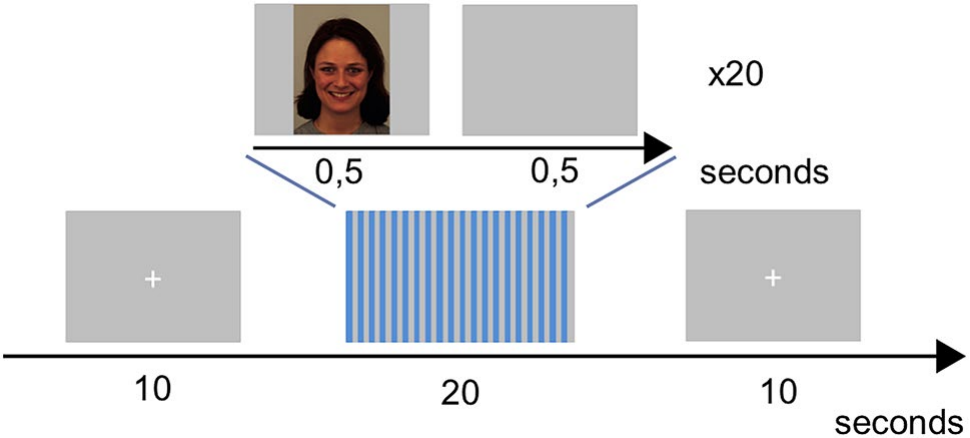
Experimental paradigm (adapted from Nilsonne et al. 2016). Between every set of three, participants were asked to rate how happy they felt and how angry they felt, using a visual analogue scale ranging from 0 to 100. The face shown in the figure is one of the stimuli (AF01HAS) from the KDEF data base that was used in the experiment^55^. Written consent exists for the publication of KDEF sample images in scientific papers; see https://www.kdef.se/home/using%20and%20publishing%20kdef%20and%20akdef.html.

MRI scanning took place in evening following the sleep intervention and control nights, starting between 5 and 8 p.m. To investigate facial emotional mimicry, participants were presented with images of facial expressions (happy, angry, or neutral), presented in blocks of 20 images (Fig. 1), using the Presentation software (Neurobehavioural Systems, Berkeley, CA, USA) through goggles (NordicNeuroLab, Bergen, Norway) adjusted for participants’ vision. Each block contained images with the same emotion, and blocks were arranged in sets of three: happy-neutral-happy or angry-neutral-angry. Each block contained 20 images and each stimulus was shown for 0.5 s with a 0.5 s inter-stimulus interval. In total, participants were presented with 12 blocks of images (4 happy, 4 angry and 4 neutral). In this manner, the total number of images with each expression was balanced over the experiment. Between every set of three blocks (i.e. 4 times in total), participants were asked to rate how happy they felt and how angry they felt, using a visual analogue scale ranging from 0 to 100 using a hand-held response box. The questions read “How happy do you feel?” and “How angry do you feel?” respectively. The cursor was initially shown at 0 and the scale was shown until response. The final number of participants for ratings of happiness and anger was 43 young and 34 older participants. Images were from the Karolinska Directed Emotional Faces (KDEF) stimulus set^55^. The same set of stimuli were used for both sleep conditions, but the order of presentation was counter-balanced across participants and conditions. Code for stimulus presentation is available at https://doi.org/10.5281/zenodo.235595. Additionally, participants provided ratings of positive and negative affect, presented in the Supplementary Information.

### Electromyography (EMG)

EMG was used to measure facial mimicry, i.e. muscle activity corresponding to smiling (major zygomatic muscle) and frowning (superciliary corrugator muscle) in response to emotional faces^13^. As reported in Nilsonne et al.,^45^ EMG was recorded using pre-gelled circular 1 cm-diameter radiotranslucent electrodes on 3.8 cm circular vinyl backing. Electrodes were placed over the major zygomatic and superciliary corrugator muscles according to established guidelines^56^. Radiotranslucent clip leads were connected through a patch panel connector to Biopac EMG amplifiers in the control room. Following a recently proposed method to remove scanner noise^57^, EMG signals were processed using a comb band stop filter with a base frequency corresponding to the number of slices/TR. Furthermore, a 30–300 Hz band pass filter was applied to exclude electrical activity not originating in muscle, and a 49–51 Hz band stop filter was used to remove line frequency noise. Zygomatic and corrugator signals were orthogonalised onto one another in order to remove overbleed and shared signal artifacts. EMG responses were binned into 1-s intervals during stimulus presentation. We rejected 3 recordings due to missing reference signals for event onsets. Data were inspected, and excessively noisy recordings after orthogonalisation were discarded (zygomatic channel: n = 5, corrugator channel: n = 30). The final sample for analysis of intervention was, for the zygomatic channel, 124 recordings from 67 participants (38 younger and 29 older), and for the corrugator channel, 103 recordings from 64 participants (37 younger and 28 older).

### MRI acquisition

We used a General Electric Discovery 3 T MRI scanner with an 8-channel head coil. Echo-planar images were acquired using the following settings: flip angle 75, TE 30, TR 2.5 s, field of view 28.8 cm, slice thickness 3 mm, 49 slices, interleaved bottom → up. This sequence was chosen to optimise imaging of the amygdala and did not give whole-brain coverage. As described in Ref.^19^, participants with less than 90% amygdala coverage were not included in this sample. The lowest slice was placed at the superior border of the pons. Five dummy volumes were run before recording began. We used higher-order shimming, where the volume of interest was restricted to a manually defined ellipsoid covering approximately the cerebrum. T1-weighted anatomical scans were acquired with a sagittal BRAVO sequence, field of view 24 cm, slice thickness 1 mm, flip angle 11, inversion time 450, interleaved bottom → up.

### fMRI preprocessing

Data were preprocessed in SPM12. Functional images were slice time corrected, realigned, normalized using DARTEL^58^ and spatially smoothed with a 8 × 8 × 8 mm kernel. One participant was excluded due to excessive head motion and one due to failed normalisation. One subject was excluded due to large signal loss. To reduce the risk of motion confounding, realignment parameters were carried forward as regressors of no interest in 1st-level analyses. Quality control was performed by manual inspection of raw structural and functional images, of segmentation results, and of head motion time courses. The final functional mask did not have full-brain coverage; it may be viewed at https://neurovault.org/collections/RLWUZRQN/images/54122/.

### fMRI analysis

1st level analyses were performed in SPM12 using linear regression at each voxel, using generalized least squares with a global approximate AR(1) autocorrelation model. The following contrasts were modelled: happy vs neutral, angry vs neutral, happy vs angry, happy versus implicit baseline (IB), i.e. blank screen, fixation cross etc., angry vs IB, neutral vs IB, happy and angry vs IB, and happy, neutral, and angry vs IB, contrasts including an implicit baseline were ad hoc analyses to investigate possible effects related to viewing of faces in general.

2nd level analyses were performed in SPM12 using linear regression at each voxel, using generalized least squares with a global repeated measures correlation model. To confirm the presence of expected main effects from the experimental paradigm, we first performed whole-brain analyses.

The main analyses focused on regions of interest (ROI:s) specified in the study preregistration. Amygdalae were defined using the Jülich atlas^59^. Fusiform face area ROI:s were defined based on Henson and Mouchlinaitis^60^ as spheres with 6 mm diameter centered on top coordinates. All ROI:s can be visualised and downloaded from https://neurovault.org/collections/RLWUZRQN/. Mean contrast estimates were determined for each region of interest and contrast of interest [happy vs neutral, angry vs neutral, happy vs angry, happy versus implicit baseline (IB), i.e. blank screen, fixation cross etc., angry vs IB, neutral vs IB, happy and angry vs IB, and happy, neutral, and angry vs IB] and were analysed using mixed-effects models in R, see below.

As secondary analyses, we investigated whole-brain effects of sleep deprivation and age group. We conducted a paired *t* test comparing the full sleep and sleep deprived conditions across both age groups and a two sample *t* tests to compare younger and older subjects.

Connectivity was investigated through a psycho-physiological interaction (PPI) approach. Seeds were based in bilateral amygdalae on the top coordinates from the contrast happy, neutral, and angry > baseline in the full sleep condition, with a 6 mm sphere around. Time courses for the BOLD signal were extracted from these seeds and entered into PPI models with the contrast happy, neutral, and angry > baseline and the PPI interaction term at first level. At second level one sample t tests were performed to investigate any change in connectivity related to viewing faces for the two sleep conditions separately. No regions displayed a significant increase in connectivity from amygdala in response to seeing faces and therefore no further analyses of connectivity were performed, since the paradigm was not judged sensitive enough to capture changes in connectivity.

### Pupil diameter and heart rate

Pupil diameter and heart rate were measured during the experiment as a measure of autonomic activity. Eye-blinks and noise were removed, as described in detail in the supplement and an average response of pupil height and width was analysed for each type of block. Heart rate was determined based on recorded pulse events and time courses were inspected for each participant and recordings judged as excessively noisy were excluded. An average response per block was calculated. Details about the analyses of pupil responses and heart rate, as well as the results from these analyses are presented in the supplement.

### Statistical methods

For ratings of happiness and anger, one mixed effects model was fitted for each type of emotion (happiness/anger) as the dependent variable, with block type, sleep condition and age group as fixed effects (allowed to interact) and a random intercept for each participant, with session nested within participant. For EMG responses, random intercepts were specified for each block, nested in session, nested in participant. For analyses of ROI data, we built separate models for each ROI contrast value estimate of interest which was included as a dependent variable and sleep condition and age group as fixed effects. A random intercept was included for each participant. Mixed-effects models were used in R^61^ with the nlme package^62^. For all mixed-effects models, contrasts were deviation coded. Where preregistered hypotheses predicted directional effects, one-sided *p* values are reported. We used a statistical significance threshold of α = 0.05 throughout because this is a conventional threshold and because we did not have sufficiently quantifiable prior probabilities to differentiate significance thresholds between different hypotheses under investigation. However, we have indicated which of the analyses that represent post-hoc analyses in the tables. All script for analyses of EMG, ratings, heart rate and pupil diameter can be found at: https://doi.org/10.5281/zenodo.3821968.

## Results

### Participants

The final number of participants available for analyses of fMRI data was 38 young and 34 older participants (for intervention effects 36 young and 33 older participants were included). Demographics are given in Table 1. Results from polysomnography recordings are previously published in Ref.^40^ but the information regarding sleep for the current sample is provided in Table 1. As previously reported, sleep deprivation caused a significant increase in self-rated sleepiness^40,46^.

**Table 1:** Participant characteristics and sleep measures. Sleep measures are shown in minutes except for sleep efficiency that is shown as %. *SD* standard deviation, *BMI* Body Mass Index, *ISI* Insomnia Severity Index, *HADS* Hospital Anxiety and Depression.

	Young	Older
n	38	34
Age, median (range)	23 (20–29)	68 (65–75)
Sex, n male, n female	17, 21	15, 19
BMI at first scanning (mean, SD)	22.6 (3.3)	24.7 (3.6)
Completed primary education (n)	0	3
Completed secondary education (n)	9	15
Currently enrolled in tertiary education (n)	25	1
Completed tertiary education (n)	4	15
ISI (mean, SD)	10.6 (2.1)	9.1 (1.5)
HADS-Depression (mean, SD)	1.1 (1.4)	1.1 (1)
Total sleep time (min), full sleep (mean, SD)	440.2 (75.1)	400.3 (62.3)
Total sleep time (min), partial sleep deprivation (mean, SD)	180 (23.9)	158.8 (32.5)
NREM (min), full sleep (mean, SD)	353 (58.7)	321.6 (61.6)
NREM (min), partial sleep deprivation (mean, SD)	154.5 (23.1)	132.8 (29.7)
N3 (min), full sleep (mean, SD)	102.9 (33.1)	44.6 (33.9)
N3 (min), partial sleep deprivation (mean, SD)	70.5 (16.7)	27.2 (24.8)
N2 (min), full sleep (mean, SD)	187.8 (50.8)	187.6 (45)
N2 (min), partial sleep deprivation (mean, SD)	65.6 (23)	72.8 (22.6)
N1 (min), full sleep (mean, SD)	62.3 (29.9)	89.5 (36.6)
N1 (min), partial sleep deprivation (mean, SD)	18.4 (9.7)	32.8 (13.6)
REM sleep (min), full sleep (mean, SD)	87.2 (30.4)	78.7 (35.)
REM sleep (min), partial sleep deprivation (mean, SD)	25.5 (12.2)	26 (18.8)
Sleep efficiency (%), full sleep (mean, SD)	93.6 (4.2)	85.8 (9.2)
Sleep efficiency (%), partial sleep deprivation (mean, SD)	94.7 (3.8)	85.7 (11.8)
Sleep latency (min), full sleep (mean, SD)	9 (8.4)	10.4 (10.1)
Sleep latency (min), partial sleep deprivation (mean, SD)	5.2 (4.4)	9.9 (15.6)
N3 latency (min), full sleep (mean, SD)	17.9 (7.3)	50.4 (65.5)
N3 latency (min), partial sleep deprivation (mean, SD)	13.2 (3.1)	33.9 (31.6)
REM latency (min), full sleep (mean, SD)	109.1 (46.1)	82.1 (40.4)
REM latency (min), partial sleep deprivation (mean, SD)	83.7 (34.5)	63.1 (32.3)

### Rated emotional experience

Participants rated their happiness and anger after blocks of angry and happy faces on a visual analogue scale (0–100). We analysed responses in a mixed-effects regression model with block type, deprivation condition, and age group as fixed effects, and a random-effects intercept for each participant. Happy stimulus blocks caused increased rated happiness compared to angry stimulus blocks (20.6 [95% CI 18.0, 23.2], *p* < 0.0001) and decreased rated anger compared to angry stimulus blocks (− 13.7 [95% CI − 16.2, − 11.1], *p* < 0.0001), as expected, all numbers representing effect estimates in original units with 95% CI.

**Figure 2:**
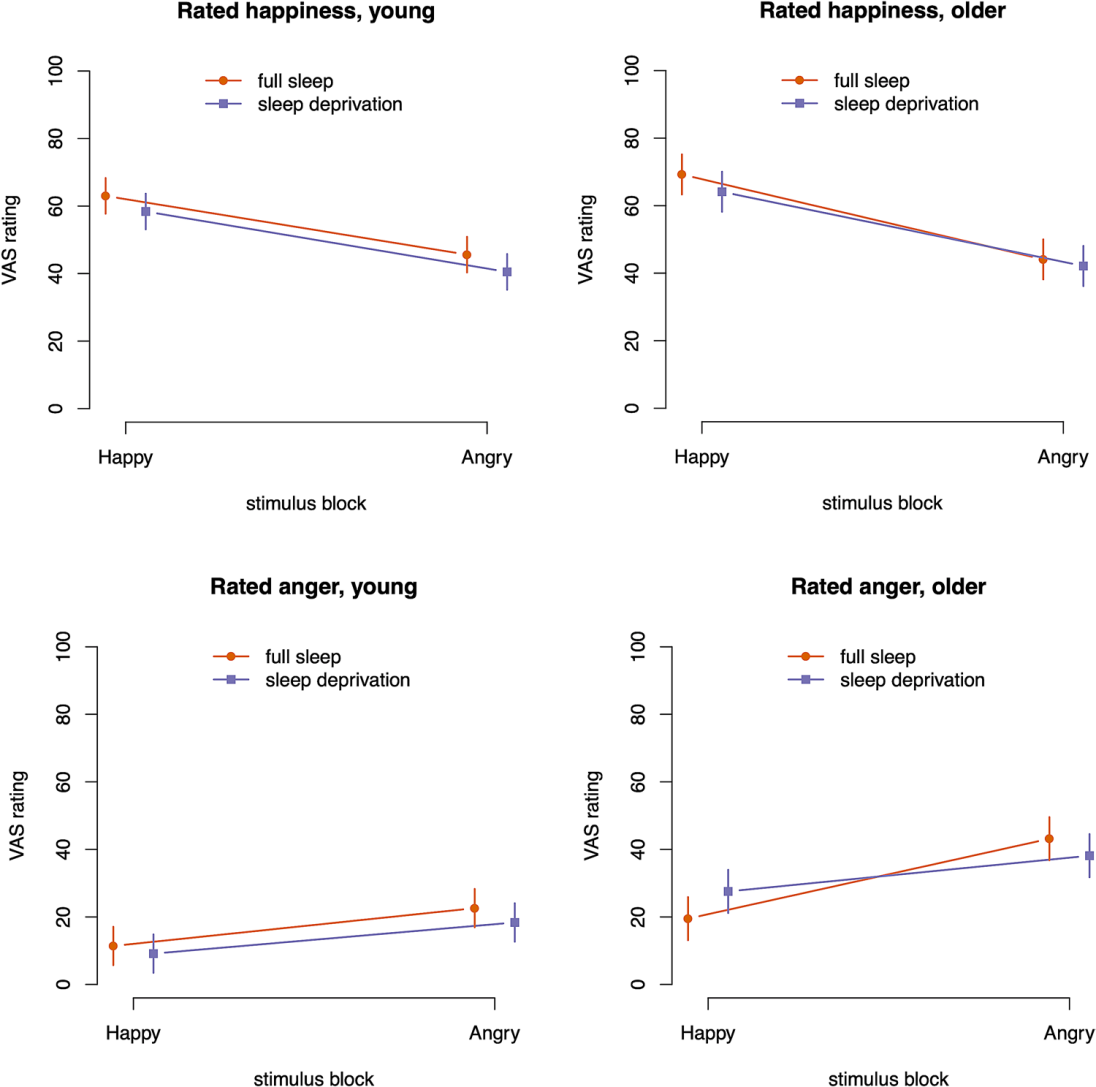
Rated emotional experience, model estimates with 95% confidence estimates.

The main effect of sleep deprivation on rated happiness was − 4.2 [− 8.3, − 0.1], *p* = 0.02 (one-sided), confirming the hypothesis that sleep deprivation would cause lower ratings of happiness. The main effect of older age group was 3.0 [− 2.8, 8.9], *p* = 0.31, and the interaction between sleep deprivation and age group was 1.3 [− 6.8, 9.5], *p* = 0.74 (Fig. 2).

The main effect of sleep deprivation on rated anger was − 0.9 [− 5.5, 3.8], *p* = 0.71, failing to support the hypothesis that sleep deprivation would cause higher ratings of anger. The main effect of older age group on rated anger was 16.7 [10.3, 23.0], *p* < 0.0001; and the interaction between sleep deprivation and age group was 4.8 [− 4.5, 14.0], *p* = 0.31 (Fig. 2). Analyses of habituation of ratings and possible covariates to the ratings are presented in Supplementary Information.

### Mimicry

As reported in Nilsonne et al.,^45^ the effect of happy faces on EMG activity across conditions and age groups over the major zygomatic muscle was 0.0047 µV [0.0001, 0.0093], p = 0.02 (one-sided), and over the corrugator muscle was − 0.0217 µV [− 0.0657, 0.0222], p = 0.17 (one-sided). The effect of angry faces on EMG activity over the major zygomatic muscle was − 0.0002 µV [− 0.0048, 0.0044], p = 0.47 (one-sided), and over the corrugator muscle was 0.0255 [− 0.0185, 0.0695], p = 0.13 (one-sided). These results are consistent with detection of emotional mimicry by EMG during fMRI scanning with this paradigm, particularly for the major zygomatic muscle in response to happy faces.

**Figure 3:**
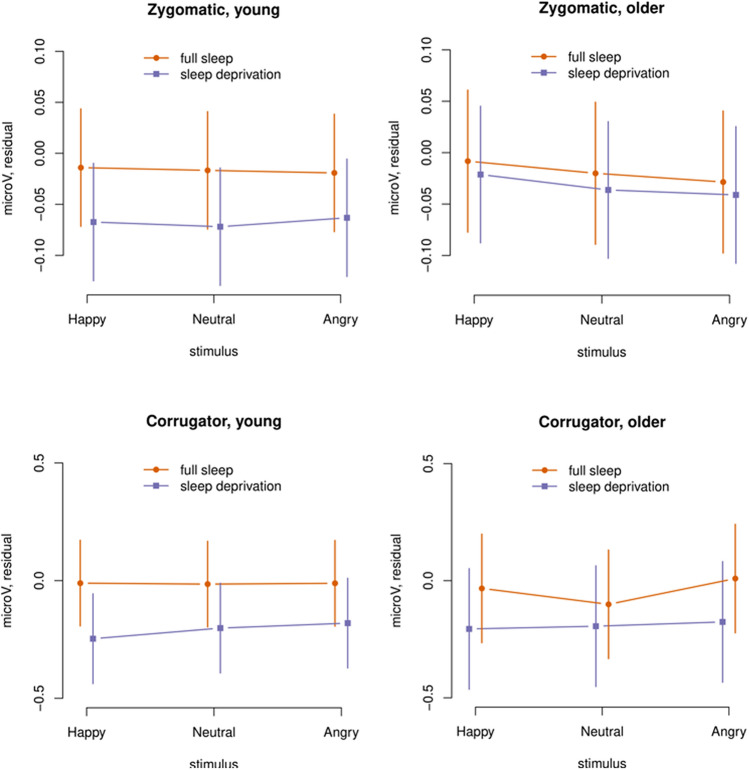
EMG activity, model estimates with 95% confidence estimates.

In a mixed-effects model with stimulus (happy, neutral, angry), condition (sleep deprivation, full sleep) and age group (young, older) as fixed effects, no strong effects were seen for zygomatic responses except a main effect of happy faces (0.0057 µV [0.0006, 0.0108], *p* = 0.015 (one-sided); Fig. 3). For corrugator responses, no major effects of sleep deprivation nor of age group were seen. The main effect of angry faces was 0.0254 µV [− 0.0075, 0.0585], *p* = 0.065. Thus, partial sleep deprivation did not affect facial muscle activity in response to emotional or neutral faces. Analyses of habituation of EMG responses and possible covariates to the responses are presented in Supplementary Information.

### fMRI main effects of conditions

**Figure 4:**
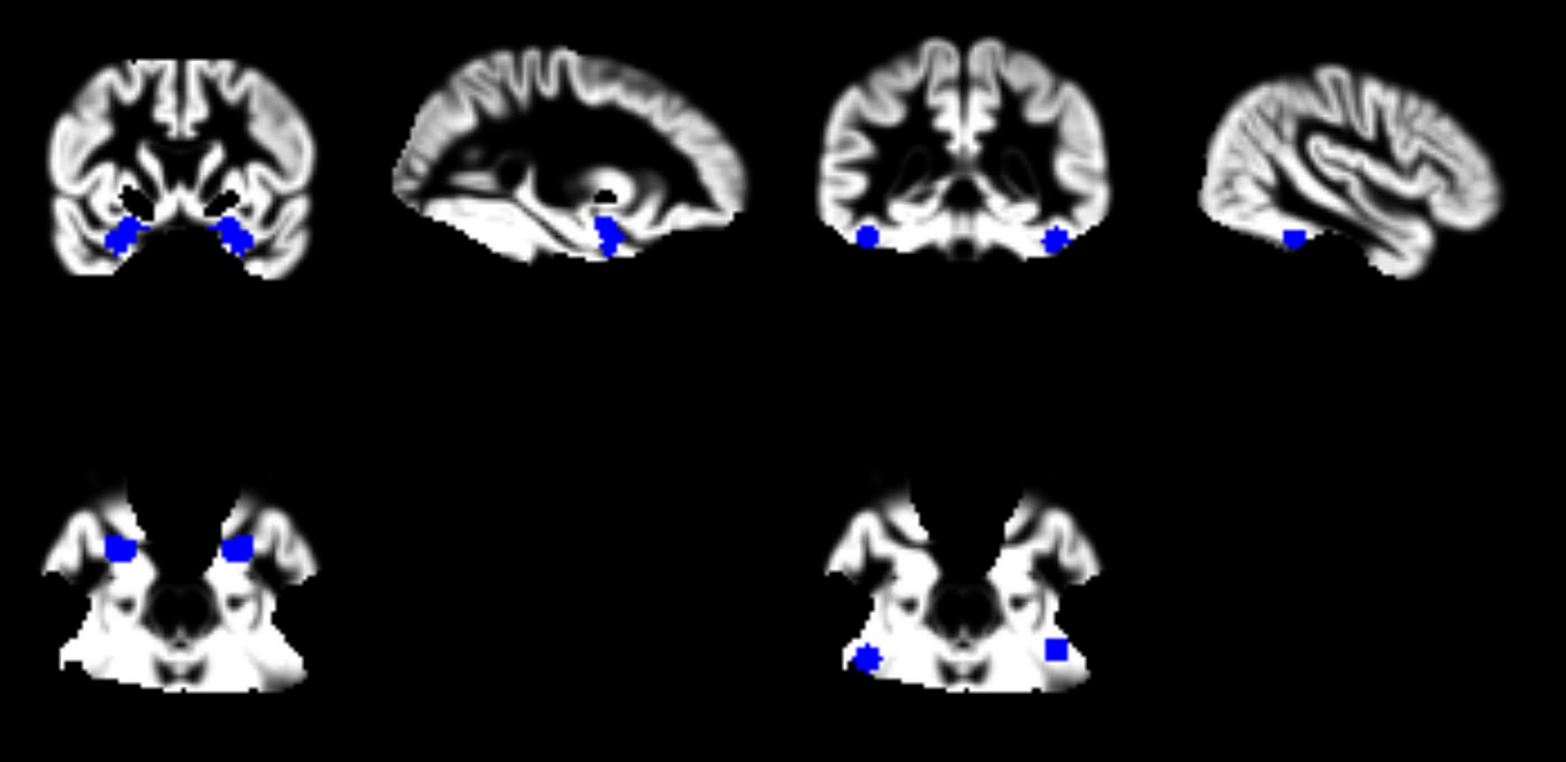
Regions of interest, shown on gray matter mask including only voxels with complete coverage across all participants. Left: amygdala ROI:s from Jülich atlas. Right: fusiform face area ROI:s from Henson and Mouchlinaitis 2007; spherical centered on top coordinates. ROI:s can be visualised and downloaded from https://neurovault.org/collections/RLWUZRQN/.

We investigated activation of the amygdala and fusiform gyrus in response to emotional faces. Regions of interest are shown in Fig. 4. We found that angry faces caused increased activation in the fusiform gyrus compared to neutral and happy faces (Table 2) whereas no strong effect was observed in the amygdala (Table 3). As a follow-up analysis focusing on the fusiform gyrus, we also compared the different emotional expressions to implicit baseline, finding strong effects of all contrasts (Table 2). As a post-hoc exploratory analysis, we investigated the same contrasts for the amygdala also, finding similarly strong effects of all contrasts (Table 3). Whole-brain results are reported in Supplementary Table 1.

**Table 2:** BOLD responses in Fusiform gyrus.

	Intercept [95% CI]	*p*	Sleep deprivation [95% CI]	*p*	Older vs younger [95% CI]	*p*	Sleep deprivation-age group interaction [95% CI]	*P*
**Fusiform (left)**								
**Happy vs angry**	**− 0.106 [− 0.205; − 0.008]**	**0.035**	**0.097 [− 0.1; 0.294]**	**0.329**	**− 0.032 [− 0.229; 0.165]**	**0.744**	**− 0.181 [− 0.575; 0.213]**	**0.362**
**Happy vs neutral**	**0.037 [− 0.057; 0.132]**	**0.216**^a^	**− 0.036 [− 0.201; 0.129]**	**0.667**	**0.021 [− 0.168; 0.21]**	**0.828**	**0.048 [− 0.283; 0.378]**	**0.775**
**Angry vs neutral**	**0.144 [0.064; 0.224]**	**< 0.001**^**a**^	**− 0.133 [− 0.293; 0.027]**	**0.103**^**b**^	**0.053 [− 0.107; 0.213]**	**0.512**	**0.229 [− 0.092; 0.549]**	**0.159**
Happy vs baseline	0.788 [0.649; 0.927]	< 0.001	0.029 [− 0.144; 0.203]	0.736	0.157 [− 0.121; 0.435]	0.264	− 0.003 [− 0.349; 0.344]	0.988
Angry vs baseline	0.894 [0.758; 1.03]	< 0.001	− 0.068 [− 0.239; 0.104]	0.434	0.189 [− 0.082; 0.461]	0.168	0.178 [− 0.164; 0.521]	0.303
Neutral vs baseline	0.75 [0.648; 0.853]	< 0.001	0.065 [− 0.068; 0.199]	0.334	0.136 [− 0.069; 0.342]	0.19	− 0.05 [− 0.318; 0.217]	0.709
Happy and angry vs baseline	1.682 [1.419; 1.944]	< 0.001	− 0.038 [− 0.299; 0.223]	0.771	0.347 [− 0.178; 0.871]	0.192	0.176 [− 0.346; 0.697]	0.504
All vs baseline	2.432 [2.081; 2.783]	< 0.001	0.027 [− 0.329; 0.383]	0.88	0.483 [− 0.219; 1.185]	0.174	0.125 [− 0.587; 0.838]	0.727
**Fusiform (right)**								
**Happy vs angry**	**− 0.063 [− 0.161; 0.036]**	**0.209**	**0.185 [− 0.012; 0.382]**	**0.065**	**− 0.024 [− 0.221; 0.173]**	**0.811**	**0.002 [− 0.392; 0.395]**	**0.993**
**Happy vs neutral**	**0.046 [− 0.048; 0.14]**	**0.168**^**a**^	**− 0.014 [− 0.194; 0.167]**	**0.88**	**0.037 [− 0.152; 0.225]**	**0.698**	**0 [− 0.361; 0.361]**	**0.999**
**Angry vs neutral**	**0.108 [0.027; 0.19]**	**0.005**^**a**^	**− 0.199 [− 0.353; − 0.045]**	**0.012**^**b**^	**0.06 [− 0.102; 0.223]**	**0.461**	**− 0.001 [− 0.309; 0.306]**	**0.993**
Happy vs baseline	1.243 [1.107; 1.379]	< 0.001	0.041 [− 0.127; 0.208]	0.627	0.007 [− 0.265; 0.278]	0.961	− 0.015 [− 0.35; 0.32]	0.93
Angry vs baseline	1.306 [1.161; 1.45]	< 0.001	− 0.144 [− 0.297; 0.009]	0.064	0.03 [− 0.259; 0.32]	0.835	− 0.016 [− 0.322; 0.289]	0.915
Neutral vs baseline	1.197 [1.073; 1.321]	< 0.001	0.055 [− 0.091; 0.2]	0.456	− 0.03 [− 0.278; 0.218]	0.809	− 0.015 [− 0.306; 0.276]	0.918
Happy and angry vs baseline	2.548 [2.284; 2.813]	< 0.001	− 0.103 [− 0.35; 0.143]	0.406	0.037 [− 0.492; 0.566]	0.889	− 0.031 [− 0.524; 0.462]	0.9
All vs baseline	3.746 [3.371; 4.12]	< 0.001	− 0.049 [− 0.394; 0.297]	0.779	0.007 [− 0.741; 0.755]	0.985	− 0.046 [− 0.737; 0.644]	0.894

Fusiform gyrus BOLD responses (contrast estimates). Analyses of pre-specified hypotheses are presented in bold and post hoc analyses in normal weight.

^a^One-sided p-value due to directional hypothesis.

^b^Two sided p-value in spite of directional hypothesis, as effect was in the contrary direction.

**Table 3:** BOLD responses in Amygdala.

Amygdala (composite)								
	Intercept [95% CI]	*p*	Sleep deprivation [95% CI]	*p*	Older vs younger [95% CI]	*p*	Sleep deprivation-age group interaction [95% CI]	*p*
**Happy vs angry**	**− 0.025 [− 0.129; 0.079]**	**0.631**	**0.183 [− 0.025; 0.391]**	**0.083**	**− 0.082 [− 0.29; 0.125]**	**0.432**	**0.006 [− 0.41; 0.421]**	**0.979**
**Happy vs neutral**	**0.037 [− 0.048; 0.122]**	**0.193**^a^	**0.049 [− 0.106; 0.205]**	**0.529**	**0.001 [− 0.169; 0.171]**	**0.991**	**0.016 [− 0.295; 0.327]**	**0.918**
**Angry vs neutral**	**0.062 [− 0.025; 0.15]**	**0.081**^a^	**− 0.134 [− 0.309; 0.041]**	**0.13**^**b**^	**0.084 [− 0.091; 0.258]**	**0.343**	**0.011 [− 0.339; 0.361]**	**0.949**
Happy vs baseline	0.337 [0.255; 0.418]	< 0.001	0.104 [− 0.059; 0.266]	0.208	− 0.156 [− 0.319; 0.006]	0.059	− 0.082 [− 0.408; 0.243]	0.616
Angry vs baseline	0.362 [0.282; 0.442]	< 0.001	− 0.08 [− 0.24; 0.081]	0.325	− 0.074 [− 0.234; 0.086]	0.36	− 0.088 [− 0.408; 0.233]	0.587
Neutral vs baseline	0.3 [0.226; 0.374]	< 0.001	0.055 [− 0.082; 0.191]	0.425	− 0.158 [− 0.305; − 0.01]	0.036	− 0.099 [− 0.372; 0.174]	0.47
Happy and angry vs baseline	0.699 [0.571; 0.827]	< 0.001	0.025 [− 0.214; 0.263]	0.837	− 0.231 [− 0.487; 0.024]	0.076	− 0.171 [− 0.649; 0.306]	0.476
All vs baseline	0.998 [0.823; 1.174]	< 0.001	0.08 [− 0.252; 0.411]	0.633	− 0.389 [− 0.741; − 0.037]	0.031	− 0.27 [− 0.933; 0.392]	0.418

Amygdala BOLD response (contrast estimates). A composite response variable was constructed by averaging right and left amygdala responses because of their high correlation (Supplementary Table 2). Analyses of pre-specified hypotheses are presented in bold and post hoc analyses in normal weight.

^a^One-sided p-value due to directional hypothesis.

^b^Two sided p-value in spite of directional hypothesis, as effect was in the contrary direction.

### fMRI effects of partial sleep deprivation and age group

Contrary to our hypothesis, partial sleep deprivation did not cause increased BOLD activation to angry faces, compared to neutral faces, in the fusiform gyrus nor in the amygdalae, using ROI-based analyses. Instead, effects were in the direction of a decrease in all three comparisons, and statistically significant for the right fusiform gyrus (Tables 2, 3). We observed no notable effects of partial sleep deprivation on activation to happy faces (Tables 2, 3). We also did not observe notable effects of age group, nor notable interactions between partial sleep deprivation and age group (Tables 2, 3). Whole-brain analyses showed no effect of partial sleep deprivation (Supplementary Table 3). For the contrasts angry > neutral and happy > neutral, younger and older participants did not show any significant differences. Age comparisons for all contrasts are presented in Supplementary Table 4.

### Data availability

Behavioural data, including EMG, heart rate and pupil diameter are available within the Zenodo repository: https://doi.org/10.5281/zenodo.3821968^63^. Statistical images of main analyses can be found at https://neurovault.org/collections/RLWUZRQN/^64^. Raw imaging data can be found at: https://openneuro.org/datasets/ds000201/^65^.

## Discussion

In the present study, effects of partial sleep deprivation on emotional contagion and mimicry were investigated using ratings of subjective emotional experience, EMG and fMRI. Partial sleep deprivation caused decreased ratings of happiness without increased ratings of anger in response to emotional faces, partly consistent with a negativity bias^7^. Contrary to our hypothesis, partial sleep deprivation did not cause an increase in amygdala and fusiform gyrus activity in response to angry compared to neutral faces. Instead, partial sleep deprivation was associated with less activation in the fusiform gyrus (significant for right side) and lower, but not significantly less, activation in the amygdala, for angry compared to neutral faces. No effect of sleep restriction on BOLD responses to happy compared to neutral faces was seen. EMG responses in the zygomatic muscle showed an effect of happy faces, confirming mimicry, but no effect of sleep restriction was seen on facial responses. Thus, the results indicate that there was a shift in emotion towards less positivity after partial sleep deprivation, but do not support the idea of increased amygdala reactivity as the mechanism.

As noted above, the hypothesis that partial sleep deprivation would cause decreased ratings of happiness and increased ratings of anger, was confirmed for ratings of happiness, but not for anger. The findings are in line with the previously reported negativity bias, with sleep deprivation being shown to primarily affect the response to positive and ambiguous stimuli, that are perceived as more negative^7^. The results are also consistent with a previous study, in which sleep deprivation was associated with lower intensity ratings of happy and angry faces^6^. The lack of effect on rated anger do not support an overall increased emotional reactivity after sleep deprivation, suggested in one previous study comparing staying awake to napping^66^.

The second hypothesis, that partial sleep deprivation would cause decreased activity in *M. corrugator* and in *M. zygomaticus* during exposure to both happy and angry faces, i.e. a less sensitive facial feedback system, could not be confirmed. To our knowledge, no previous study has examined the effect of sleep deprivation on emotional mimicry using both EMG and fMRI. However, it has previously been reported that sleep deprivation was associated with fewer facial movements to sad and amusing film clips judged by other people (not EMG)^38^ but more frowning was seen after wakefulness compared to napping^67^. Also, slower facial (EMG) responses to positive and negative stimuli, including happy and angry faces^4^ were reported after a partial sleep deprivation condition compared to a control. The data in the present study were filtered to remove scanner noise. It can therefore not be excluded that some small effects might have been masked during the acquisition or filtering. However, the strong effect of happy faces indicates that EMG during MRI-scanning could indeed be used to study emotional mimicry, and the results indicate that emotional facial mimicry is stable to partial sleep deprivation.

The third hypothesis concerned the effect of partial sleep deprivation on the BOLD responses in the amygdala and the fusiform gyrus in response to the emotional faces. As hypothesised, angry faces compared to neutral faces caused an increased activity in the fusiform gyrus and a trend significant increase in the amygdala. Unexpectedly, partial sleep deprivation was associated with a decrease in activity in all these areas for the contrast angry vs neutral, but significant only in the right fusiform gyrus. For happy faces compared to neutral, there was no significant increase in activity neither in amygdala nor the fusiform gyrus, and partial sleep deprivation was not associated with any change in the response. The present results stand in contrast to what was found in the studies by Motomura et al.^31^ and Yoo et al.^27^ A potential shortcoming of the present study was the overall lack of effect in amygdala for emotional versus non-emotional faces, which might question the validity of the paradigm for investigating effects on the amygdala. However, previous studies indicate that also non-emotional faces can cause an increase in amygdala activity^68–70^, and in the present study there was indeed a highly significant effect of all faces compared to baseline in the amygdala. Another important difference between the present study and the work by Yoo and Motomura^27,31^ is the use of partial sleep deprivation as compared to total sleep deprivation, with potentially weaker effects. Some studies have also highlighted the role of REM-sleep for emotional recovery^29,71^ and allowing participants to sleep in the end of the night might have been sufficient for them to obtain enough of this specific sleep stage. Regardless of which part of sleep that impact reactions to emotional stimuli, a dose–response effect could be likely, explaining the difference between the present study and studies using total sleep deprivation or repeated nights of restricted sleep. However, as mentioned above, the direction of the effects in amygdala and fusiform gyrus was towards a decrease in activity after partial sleep deprivation, i.e. the opposite direction to the previous studies^27,31^. As noted in the introduction, the literature on sleep deprivation and emotional functions show some inconsistencies and further work is needed to disentangle potential modifiers of the response as well as to replicate previous findings. Despite the already discussed previous studies showing increased amygdala reactivity to negative stimuli, including fearful faces, after sleep deprivation^27,31^, the previous evidence of increased amygdala reactivity and inhibited top-down control after sleep deprivation, is still limited. There is therefore a specific need for large studies with careful monitoring of sleep.

The present study is to our knowledge the first study showing an effect of sleep deprivation in the fusiform gyrus to emotional faces. As described in the introduction, the fusiform gyrus is not primarily involved in the emotional aspects of face processing^23^. The fusiform face area has however been shown to have higher within-subject reliability than amygdala for face processing^72^. The effect of partial sleep deprivation was seen for the contrast angry vs neutral faces, possibly indicating a specificity for emotion. The findings raise the possibility that the effect of sleep deprivation on emotional processing might be partly dependent on neural structures that are also involved in more general aspects of face processing, such as the fusiform gyri. However, further studies need to replicate this finding.

A secondary aim in this study was to investigate adult age differences in the context of emotional contagion and mimicry, as well as the response to partial sleep deprivation. In summary, there was no significant differences between younger and older subjects for emotional faces vs neutral, or for the effect of partial sleep deprivation. We have previously shown that there might be differences in how younger and older individuals respond to sleep deprivation for mood and empathy^20,39^, but the present data do not suggest this being true for emotional face processing. For the contrasts neutral vs baseline and all faces vs baseline, there was significantly less amygdala responses in older compared to young. This finding is in line with previous studies^73,74^, and could possibly be related to amygdala atrophy^74^. Older participants rated higher levels of anger in response to both happy and angry faces. This finding is unexpected and stands in contrast to the previously reported positivity effect in ageing^43,75^.

Limitations in the present study include the sample size and a potential selection bias, specifically for the older group. The sample was relatively large compared to previous studies of sleep and emotion^27,31,76^, but because the effects of interest could be expected to be small, statistical power might still be too low. As noted above, the present study also used partial sleep deprivation with potentially weaker effects, compared to total sleep deprivation. As described in previous publications from the same sample^40,46^ some of the participants slept relatively short also in the full sleep condition, further diluting any difference between the conditions. Other limitations include the limited ecological validity (i.e. photographs and not for example real human interaction) of the paradigm and the scanner noise in the EMG-signal. The latter might be of a certain importance, since the main effect of angry faces on corrugator muscle activity was not significant and effects of partial sleep deprivation on facial mimicry should be expected to be even smaller than the main effect of angry faces.

## Conclusion

In conclusion, the present study further underlines the importance of sleep for basal social emotional abilities related to sharing of emotion, but does not indicate a moderating role of adult ageing. Subjective ratings of emotional experience of less happiness are consistent with an increased negativity bias after partial sleep deprivation. However, brain responses were inconsistent with previously proposed inhibited top-down control and increased amygdala reactivity as a mechanism behind altered emotional contagion after sleep deprivation. To the contrary, the results indicate blunting of the responses, possibly reflecting an increased activation threshold in fusiform gyrus, and potentially also amygdala, as a possible neural mechanism for the behavioural effects of partial sleep deprivation on responses to emotional faces.

## Supplementary information

Supplementary Information.
