# Supplementary material for: A combined fMRI and EMG study of emotional contagion following partial sleep deprivation in young and older humans
Source: Sci Rep. 2020 Oct 21;10:17944. doi: 10.1038/s41598-020-74489-9 (PMC7578048; doi:10.1038/s41598-020-74489-9)
Supplement: Supplementary file 1 — Supplementary Information. [file 41598_2020_74489_MOESM1_ESM.docx]

**A combined fMRI and EMG study of emotional contagion following partial sleep deprivation in young and older humans**

Sandra Tamm^1,2,*^, Johanna Schwarz^1,2^, Hanna Thuné^3^, Göran Kecklund^1^, Predrag Petrovic^2^, Torbjörn Åkerstedt^1,2^, Håkan Fischer^4^, Mats Lekander^1,2^, Gustav Nilsonne^1,2^

1: Stockholm University, Department of Psychology, Stress Research Institute

2: Karolinska Institutet, Department of Clinical Neuroscience

3: University of Glasgow, Department of Psychology

4: Stockholm University, Department of Psychology

*Corresponding: sandra.tamm@ki.se

**Supplementary material**

**Main effects of stimuli**

Supplementary table 1a includes a list of main effects of stimulus types in the full sleep condition across groups (1 sample t tests). Supplementary table 1b-1g show SPM tables for the corresponding analyses and all unthresholded t maps can be downloaded from Neurovault (see table 1 for specific links). The main contrasts are also shown in supplementary fig 1, below.

**Supplementary table 1a: Overview of whole-brain results for main contrasts across groups, full sleep condition**

| **1^st^ level contrast** | ***n* clusters, *p_FWE_* < 0.05** | **Clusters listed in** | **Unthresholded *t* map** |
| --- | --- | --- | --- |
| Angry_vs_Neutral | 7 | Suppl. table 1b | <https://neurovault.org/collections/RLWUZRQN/images/54844/> |
| Happy_vs_Neutral | 1 | Suppl. table 1c | <https://neurovault.org/collections/RLWUZRQN/images/54846/> |
| Happy_vs_Baseline | 10 | Suppl. Table 1d | <https://neurovault.org/collections/RLWUZRQN/images/54848/> |
| Angry_vs_Baseline | 18 | Suppl. Table 1e | <https://neurovault.org/collections/RLWUZRQN/images/110706/> |
| Neutral_vs_Baseline | 14 | Suppl. Table 1f | <https://neurovault.org/collections/RLWUZRQN/images/110707/> |
| All_vs_Baseline | 19 | Suppl. table 1g* | <https://neurovault.org/collections/RLWUZRQN/images/110708/> |

*Also previously reported in Nilsonne 2016.


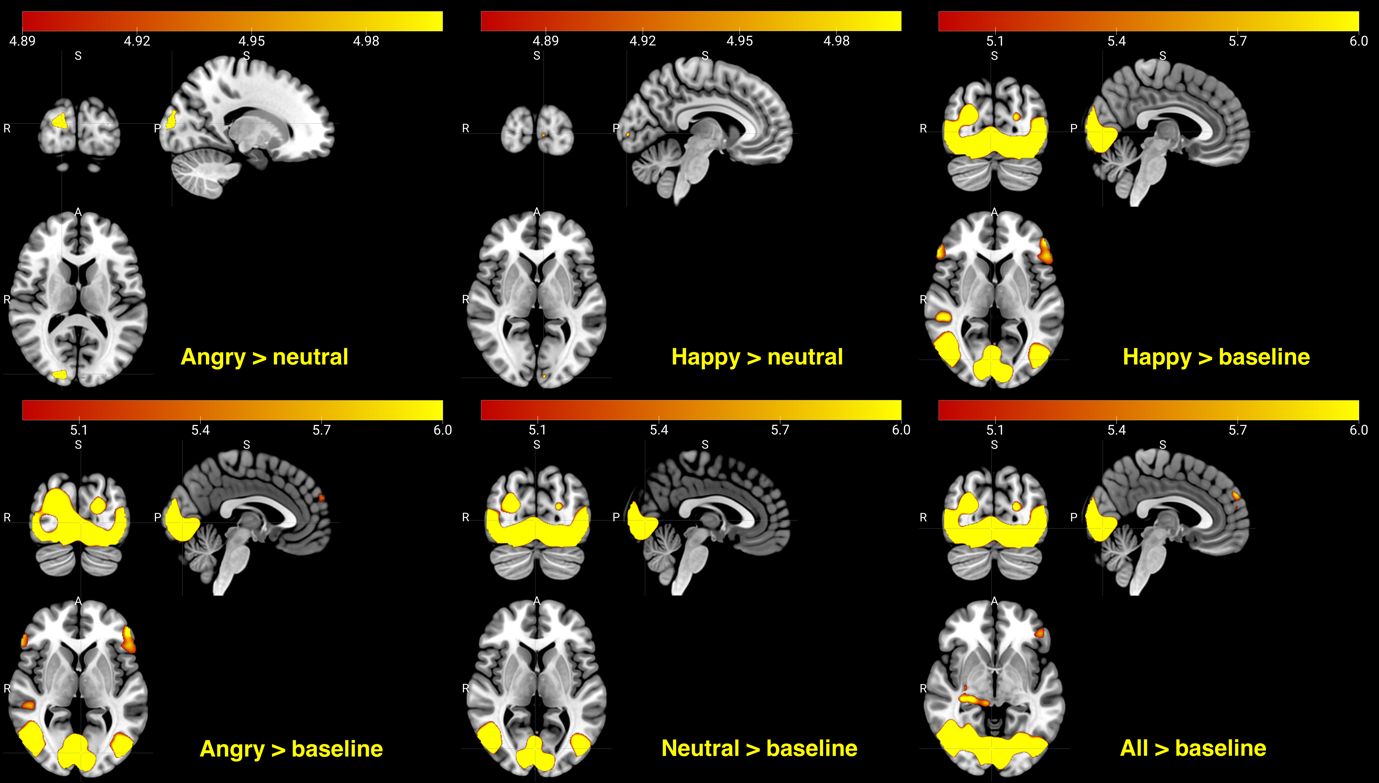


Supplementary figure 1. Overview of whole-brain results for main contrasts across groups, full sleep condition

**Supplementary table 1b: Angry vs neutral, SPM results table**

| **cluster-level** | | | | **peak-level** | | | | | **MNI (mm)** | | |
| --- | --- | --- | --- | --- | --- | --- | --- | --- | --- | --- | --- |
| ***p_FWE_*** | ***p_FDR_*** | **equiv *k*** | ***p_unc_*** | ***p_FWE_*** | ***p_FDR_*** | ***t*** | **equiv *z*** | ***p_unc_*** | **x** | **y** | **z** |
| 0 | 0.002 | 196 | 0 | 0 | 0.007 | 6.86 | 6 | 0 | 18 | -94 | 14 |
|  |  |  |  | 0.03 | 0.757 | 5.05 | 4.66 | 0 | 18 | -86 | 24 |
| 0.019 | 0.535 | 8 | 0.382 | 0.017 | 0.757 | 5.23 | 4.8 | 0 | 44 | -42 | -16 |
| 0.007 | 0.473 | 24 | 0.135 | 0.018 | 0.757 | 5.2 | 4.77 | 0 | 50 | -36 | 4 |
| 0.015 | 0.535 | 11 | 0.304 | 0.022 | 0.757 | 5.15 | 4.73 | 0 | -36 | -76 | -18 |
| 0.018 | 0.535 | 9 | 0.353 | 0.027 | 0.757 | 5.09 | 4.69 | 0 | -36 | -64 | -16 |
| 0.031 | 0.606 | 3 | 0.606 | 0.036 | 0.757 | 4.99 | 4.61 | 0 | -6 | -96 | 12 |
| 0.028 | 0.606 | 4 | 0.545 | 0.038 | 0.757 | 4.98 | 4.6 | 0 | -28 | -94 | 14 |

**Supplementary table 1c: Happy vs neutral, SPM results table**

| **cluster-level** | | | | **peak-level** | | | | | **MNI (mm)** | | |
| --- | --- | --- | --- | --- | --- | --- | --- | --- | --- | --- | --- |
| ***p_FWE_*** | ***p_FDR_*** | **equiv *k*** | ***p_unc_*** | ***p_FWE_*** | ***p_FDR_*** | ***t*** | **equiv *z*** | ***p_unc_*** | **x** | **y** | **z** |
| 0.018 | 0.345 | 10 | 0.345 | 0.027 | 0.621 | 5.06 | 4.67 | 0 | -6 | -96 | 0 |

**Supplementary table 1d: Happy vs baseline, SPM results table**

| **cluster-level** | | | | **peak-level** | | | | | **MNI (mm)** | | |
| --- | --- | --- | --- | --- | --- | --- | --- | --- | --- | --- | --- |
| ***p_FWE_*** | ***p_FDR_*** | **equiv *k*** | ***p_unc_*** | ***p_FWE_*** | ***p_FDR_*** | ***t*** | **equiv *z*** | ***p_unc_*** | **x** | **y** | **z** |
| 0 | 0 | 13234 | 0 | 0 | 0 | 21.64 | Inf | 0 | 8 | -78 | -6 |
|  |  |  |  | 0 | 0 | 21.34 | Inf | 0 | 12 | -90 | 12 |
|  |  |  |  | 0 | 0 | 20.63 | Inf | 0 | 14 | -90 | 20 |
| 0 | 0 | 401 | 0 | 0 | 0 | 8.39 | 6.98 | 0 | 22 | -6 | -14 |
|  |  |  |  | 0 | 0.003 | 6.71 | 5.89 | 0 | 32 | -14 | -12 |
|  |  |  |  | 0 | 0.014 | 6.25 | 5.57 | 0 | 32 | -6 | -16 |
| 0 | 0 | 244 | 0 | 0 | 0.001 | 6.89 | 6.02 | 0 | -20 | -6 | -16 |
| 0.001 | 0.063 | 52 | 0.025 | 0.004 | 0.1 | 5.69 | 5.15 | 0 | 54 | 4 | 46 |
| 0.01 | 0.374 | 16 | 0.187 | 0.004 | 0.105 | 5.65 | 5.12 | 0 | 22 | -28 | -2 |
| 0.012 | 0.387 | 13 | 0.232 | 0.004 | 0.105 | 5.65 | 5.12 | 0 | -12 | 46 | 50 |
| 0.019 | 0.462 | 7 | 0.38 | 0.006 | 0.14 | 5.55 | 5.05 | 0 | 34 | -6 | -34 |
| 0.015 | 0.419 | 10 | 0.294 | 0.013 | 0.28 | 5.34 | 4.89 | 0 | 42 | 6 | 30 |
| 0.021 | 0.462 | 6 | 0.418 | 0.033 | 0.694 | 5.05 | 4.66 | 0 | 0 | 60 | 18 |
| 0.023 | 0.462 | 5 | 0.462 | 0.034 | 0.694 | 5.05 | 4.66 | 0 | -2 | 66 | 18 |

**Supplementary table 1e: Angry vs baseline, SPM results table**

| **cluster-level** | | | | **peak-level** | | | | | **MNI (mm)** | | |
| --- | --- | --- | --- | --- | --- | --- | --- | --- | --- | --- | --- |
| ***p_FWE_*** | ***p_FDR_*** | **equiv *k*** | ***p_unc_*** | ***p_FWE_*** | ***p_FDR_*** | ***t*** | **equiv *z*** | ***p_unc_*** | **x** | **y** | **z** |
| 0 | 0 | 15492 | 0 | 0 | 0 | 23.26 | Inf | 0 | 12 | -90 | 12 |
|  |  |  |  | 0 | 0 | 22.86 | Inf | 0 | 10 | -78 | -6 |
|  |  |  |  | 0 | 0 | 21.09 | Inf | 0 | -14 | -94 | 16 |
| 0 | 0 | 1100 | 0 | 0 | 0 | 8.39 | 6.99 | 0 | 20 | -6 | -14 |
|  |  |  |  | 0 | 0 | 8.24 | 6.89 | 0 | 34 | -12 | -14 |
|  |  |  |  | 0 | 0 | 7.77 | 6.6 | 0 | 24 | -14 | -10 |
| 0 | 0 | 342 | 0 | 0 | 0 | 8.32 | 6.94 | 0 | -20 | -6 | -14 |
|  |  |  |  | 0.003 | 0.066 | 5.77 | 5.22 | 0 | -32 | -14 | -14 |
| 0 | 0 | 415 | 0 | 0 | 0 | 7.47 | 6.41 | 0 | 56 | 30 | 20 |
|  |  |  |  | 0.001 | 0.021 | 6.12 | 5.48 | 0 | 58 | 30 | 4 |
|  |  |  |  | 0.005 | 0.112 | 5.6 | 5.09 | 0 | 46 | 30 | 10 |
| 0 | 0 | 478 | 0 | 0 | 0 | 7.29 | 6.29 | 0 | 54 | 4 | 50 |
|  |  |  |  | 0 | 0.004 | 6.64 | 5.84 | 0 | 50 | 2 | 40 |
|  |  |  |  | 0 | 0.015 | 6.25 | 5.57 | 0 | 44 | 12 | 26 |
| 0 | 0 | 821 | 0 | 0 | 0.001 | 7.06 | 6.13 | 0 | -52 | 44 | 0 |
|  |  |  |  | 0.001 | 0.019 | 6.18 | 5.52 | 0 | -46 | 24 | -12 |
|  |  |  |  | 0.001 | 0.021 | 6.12 | 5.48 | 0 | -56 | 32 | 12 |

**Supplementary table 1e (continued)**

| 0 | 0 | 241 | 0 | 0 | 0.001 | 6.98 | 6.08 | 0 | 56 | -36 | 8 |
| --- | --- | --- | --- | --- | --- | --- | --- | --- | --- | --- | --- |
|  |  |  |  | 0 | 0.004 | 6.62 | 5.83 | 0 | 46 | -36 | 8 |
| 0 | 0 | 211 | 0 | 0 | 0.004 | 6.63 | 5.84 | 0 | -10 | 52 | 42 |
|  |  |  |  | 0 | 0.011 | 6.34 | 5.64 | 0 | -8 | 62 | 28 |
|  |  |  |  | 0.001 | 0.019 | 6.17 | 5.51 | 0 | -16 | 56 | 32 |
| 0 | 0.008 | 98 | 0.004 | 0.001 | 0.019 | 6.18 | 5.52 | 0 | -42 | -4 | 46 |
| 0.017 | 0.37 | 9 | 0.329 | 0.001 | 0.028 | 6.04 | 5.42 | 0 | -34 | 18 | -32 |
| 0.001 | 0.019 | 75 | 0.01 | 0.002 | 0.061 | 5.81 | 5.25 | 0 | 40 | -6 | -36 |
| 0.004 | 0.104 | 32 | 0.075 | 0.003 | 0.071 | 5.74 | 5.19 | 0 | 16 | 50 | 44 |
| 0.002 | 0.07 | 41 | 0.047 | 0.004 | 0.098 | 5.65 | 5.12 | 0 | -48 | 12 | 52 |
| 0.001 | 0.029 | 62 | 0.018 | 0.006 | 0.133 | 5.55 | 5.04 | 0 | -46 | 18 | 22 |
| 0.006 | 0.151 | 24 | 0.118 | 0.017 | 0.374 | 5.24 | 4.81 | 0 | 48 | 10 | -36 |
| 0.022 | 0.453 | 6 | 0.428 | 0.029 | 0.597 | 5.09 | 4.69 | 0 | 40 | 28 | -16 |
| 0.014 | 0.336 | 11 | 0.28 | 0.032 | 0.648 | 5.06 | 4.66 | 0 | 52 | 38 | -10 |
|  |  |  |  | 0.037 | 0.741 | 5.01 | 4.63 | 0 | 54 | 30 | -8 |
| 0.039 | 0.773 | 1 | 0.773 | 0.05 | 0.99 | 4.92 | 4.55 | 0 | 42 | 22 | -20 |

**Supplementary table 1f: Neutral vs baseline, SPM results table**

| **cluster-level** | | | | **peak-level** | | | | | **MNI (mm)** | | |
| --- | --- | --- | --- | --- | --- | --- | --- | --- | --- | --- | --- |
| ***p_FWE_*** | ***p_FDR_*** | **equiv *k*** | ***p_unc_*** | ***p_FWE_*** | ***p_FDR_*** | ***t*** | **equiv *z*** | ***p_unc_*** | **x** | **y** | **z** |
| 0 | 0 | 13397 | 0 | 0 | 0 | 24.66 | Inf | 0 | 10 | -78 | -6 |
|  |  |  |  | 0 | 0 | 24.13 | Inf | 0 | 10 | -90 | 10 |
|  |  |  |  | 0 | 0 | 21.86 | Inf | 0 | -10 | -94 | 14 |
| 0 | 0 | 264 | 0 | 0 | 0 | 10.06 | Inf | 0 | -18 | -6 | -16 |
| 0 | 0 | 673 | 0 | 0 | 0 | 8.31 | 6.94 | 0 | 20 | -6 | -16 |
|  |  |  |  | 0 | 0 | 7.76 | 6.59 | 0 | 32 | -12 | -12 |
|  |  |  |  | 0 | 0 | 7.75 | 6.59 | 0 | 32 | -4 | -18 |
| 0 | 0.024 | 67 | 0.009 | 0 | 0.008 | 6.52 | 5.76 | 0 | 56 | 2 | 46 |
| 0 | 0.009 | 94 | 0.003 | 0.001 | 0.022 | 6.22 | 5.54 | 0 | -48 | -2 | 56 |
|  |  |  |  | 0.003 | 0.092 | 5.77 | 5.21 | 0 | -56 | -4 | 48 |
|  |  |  |  | 0.004 | 0.118 | 5.68 | 5.15 | 0 | -62 | -8 | 40 |
| 0.011 | 0.309 | 12 | 0.221 | 0.001 | 0.032 | 6.1 | 5.46 | 0 | 34 | -8 | -34 |
| 0.003 | 0.102 | 31 | 0.058 | 0.001 | 0.034 | 6.07 | 5.44 | 0 | -36 | 32 | -14 |
| 0.001 | 0.027 | 58 | 0.013 | 0.003 | 0.081 | 5.81 | 5.25 | 0 | 44 | 6 | 32 |
| 0.001 | 0.027 | 59 | 0.013 | 0.008 | 0.194 | 5.51 | 5.02 | 0 | 48 | 30 | 14 |
| 0.016 | 0.402 | 8 | 0.316 | 0.009 | 0.207 | 5.48 | 4.99 | 0 | -38 | 24 | 52 |
| 0.011 | 0.309 | 12 | 0.221 | 0.024 | 0.514 | 5.19 | 4.77 | 0 | -14 | 58 | 30 |
| 0.038 | 0.75 | 1 | 0.75 | 0.031 | 0.637 | 5.11 | 4.7 | 0 | 44 | -38 | 10 |

**Supplementary table 1f (continued)**

| 0.038 | 0.75 | 1 | 0.75 | 0.043 | 0.886 | 5 | 4.62 | 0 | -46 | 24 | 44 |
| --- | --- | --- | --- | --- | --- | --- | --- | --- | --- | --- | --- |
| 0.038 | 0.75 | 1 | 0.75 | 0.046 | 0.911 | 4.99 | 4.61 | 0 | -18 | 40 | 50 |

**Supplementary table 1g: All vs baseline, SPM results table**

| **cluster-level** | | | | **peak-level** | | | | | **MNI (mm)** | | |
| --- | --- | --- | --- | --- | --- | --- | --- | --- | --- | --- | --- |
| ***p_FWE_*** | ***p_FDR_*** | **equiv *k*** | ***p_unc_*** | ***p_FWE_*** | ***p_FDR_*** | ***t*** | **equiv *z*** | ***p_unc_*** | **x** | **y** | **z** |
| 0 | 0 | 15276 | 0 | 0 | 0 | 25.55 | Inf | 0 | 10 | -78 | -6 |
|  |  |  |  | 0 | 0 | 25.14 | Inf | 0 | 10 | -90 | 10 |
|  |  |  |  | 0 | 0 | 22.31 | Inf | 0 | -12 | -94 | 14 |
| 0 | 0 | 583 | 0 | 0 | 0 | 11.68 | Inf | 0 | -20 | -6 | -16 |
| 0 | 0 | 1279 | 0 | 0 | 0 | 10.8 | Inf | 0 | 20 | -6 | -14 |
|  |  |  |  | 0 | 0 | 10.32 | Inf | 0 | 34 | -12 | -14 |
|  |  |  |  | 0 | 0 | 9.73 | 7.75 | 0 | 32 | -4 | -18 |
| 0 | 0.012 | 74 | 0.006 | 0 | 0 | 7.87 | 6.67 | 0 | 34 | -6 | -34 |
| 0 | 0 | 730 | 0 | 0 | 0 | 7.64 | 6.52 | 0 | 54 | 4 | 50 |
|  |  |  |  | 0 | 0.002 | 6.87 | 6.01 | 0 | 44 | 8 | 30 |
|  |  |  |  | 0 | 0.002 | 6.8 | 5.96 | 0 | 56 | 32 | 18 |
| 0 | 0 | 188 | 0 | 0 | 0 | 7.47 | 6.41 | 0 | -38 | 30 | -14 |
| 0 | 0 | 459 | 0 | 0 | 0 | 7.46 | 6.4 | 0 | -12 | 48 | 48 |
|  |  |  |  | 0 | 0.001 | 7.21 | 6.24 | 0 | -6 | 62 | 28 |

**Supplementary table 1g (continued)**

|  |  |  |  | 0 | 0.001 | 7.04 | 6.12 | 0 | -8 | 54 | 40 |
| --- | --- | --- | --- | --- | --- | --- | --- | --- | --- | --- | --- |
| 0 | 0 | 216 | 0 | 0 | 0.001 | 7.1 | 6.16 | 0 | -48 | -2 | 56 |
|  |  |  |  | 0 | 0.008 | 6.46 | 5.72 | 0 | -50 | 10 | 50 |
|  |  |  |  | 0.03 | 0.614 | 5.12 | 4.71 | 0 | -46 | -4 | 44 |
| 0 | 0.012 | 72 | 0.006 | 0 | 0.012 | 6.34 | 5.63 | 0 | 44 | -36 | 8 |
|  |  |  |  | 0.012 | 0.26 | 5.4 | 4.93 | 0 | 56 | -34 | 8 |
| 0.008 | 0.199 | 16 | 0.157 | 0 | 0.012 | 6.33 | 5.63 | 0 | -34 | 18 | -32 |
| 0 | 0.003 | 113 | 0.001 | 0.001 | 0.035 | 6.02 | 5.4 | 0 | -48 | 40 | 0 |
| 0.005 | 0.138 | 23 | 0.095 | 0.004 | 0.106 | 5.69 | 5.15 | 0 | -56 | 30 | 14 |
| 0.002 | 0.076 | 35 | 0.044 | 0.004 | 0.106 | 5.69 | 5.15 | 0 | 14 | 50 | 46 |
|  |  |  |  | 0.007 | 0.171 | 5.53 | 5.03 | 0 | 18 | 42 | 50 |
| 0.003 | 0.108 | 28 | 0.068 | 0.006 | 0.149 | 5.58 | 5.07 | 0 | -42 | 18 | 24 |
| 0.016 | 0.348 | 8 | 0.312 | 0.011 | 0.244 | 5.42 | 4.95 | 0 | 58 | 16 | 36 |
| 0.007 | 0.183 | 18 | 0.135 | 0.013 | 0.278 | 5.38 | 4.91 | 0 | 32 | 18 | -28 |
| 0.028 | 0.576 | 3 | 0.545 | 0.025 | 0.543 | 5.17 | 4.75 | 0 | 40 | 28 | -16 |
| 0.01 | 0.237 | 13 | 0.2 | 0.028 | 0.578 | 5.15 | 4.73 | 0 | 48 | 10 | -34 |
| 0.038 | 0.747 | 1 | 0.747 | 0.044 | 0.884 | 5 | 4.62 | 0 | -32 | -10 | -32 |

**Supplementary table 2: Correlations between ROI effect estimates from right and left sides.** High correlations justified the use of composite variables averaging activity on the right and left sides (for Amygdala).

| **Amygdala** | | |
| --- | --- | --- |
|  | Full sleep | Sleep deprived |
| **All_vs_Baseline** | 0.85 [0.77, 0.91] | 0.83 [0.73, 0.89] |
| **Happy_vs_Angry** | 0.87 [0.80, 0.92] | 0.86 [0.78, 0.91] |
| **Happy_vs_Neutral** | 0.85 [0.77, 0.91] | 0.87 [0.79, 0.92] |
| **Angry_vs_Neutral** | 0.85 [0.77, 0.91] | 0.88 [0.82, 0.93] |
| **Happy_vs_Baseline** | 0.83 [0.74, 0.89] | 0.74 [0.62, 0.83] |
| **Angry_vs_Baseline** | 0.78 [0.67, 0.86] | 0.84 [0.75, 0.90] |
| **Neutral_vs_Baseline** | 0.84 [0.75, 0.90] | 0.82 [0.72, 0.88] |
| **Happy_and_Angry_vs_Baseline** | 0.80 [0.70, 0.87] | 0.82 [0.72, 0.88] |
| **Fusiform gyrus** | | |
|  | Full sleep | Sleep deprived |
| **All_vs_Baseline** | 0.87 [0.80, 0.92] | 0.64 [0.48, 0.76] |
| **Happy_vs_Angry** | 0.83 [0.73, 0.89] | 0.72 [0.58, 0.82] |
| **Happy_vs_Neutral** | 0.82 [0.72, 0.88] | 0.78 [0.67, 0.86] |
| **Angry_vs_Neutral** | 0.69 [0.55, 0.80] | 0.64 [0.48, 0.76] |
| **Happy_vs_Baseline** | 0.75 [0.62, 0.83] | 0.57 [0.39, 0.71] |
| **Angry_vs_Baseline** | 0.60 [0.42, 0.73] | 0.56 [0.38, 0.71] |
| **Neutral_vs_Baseline** | 0.68 [0.53, 0.79] | 0.60 [0.43, 0.73] |
| **Happy_and_Angry_vs_Baseline** | 0.64 [0.48, 0.76] | 0.57 [0.38, 0.71] |

For Amygdala, all correlations were > 0.80 except two that were 0.78 and 0.74.

**Supplementary table 3: Whole-brain results for 2nd level contrast sleep deprivation vs full sleep**

Supplementary table 3 includes comparisons between the full sleep condition and partial sleep deprivation condition with links to the unthresholded t maps on Neurovault (paired t tests). There were no significant clusters at *p_FWE_* < 0.05.

| **1st level contrast** | **Unthresholded *t* map** |
| --- | --- |
| **Angry_vs_Neutral** | <https://neurovault.org/collections/RLWUZRQN/images/54185/> |
| **Happy_vs_Neutral** | <https://neurovault.org/collections/RLWUZRQN/images/54191/> |
| **Happy_vs_Baseline** | <https://neurovault.org/collections/RLWUZRQN/images/54192/> |
| **Angry_vs_Baseline** | <https://neurovault.org/collections/RLWUZRQN/images/54193/> |
| **Neutral_vs_Baseline** | <https://neurovault.org/collections/RLWUZRQN/images/54194/> |
| **All_vs_Baseline** | <https://neurovault.org/collections/RLWUZRQN/images/54195/> |

**Supplementary table 4: Whole-brain results for 2nd level contrast younger vs older, full sleep condition**

Supplementary table 4 includes comparisons between the younger and older participants with links to the unthresholded t maps on Neurovault (2 sample t tests).

| **1st level contrast** | **Unthresholded *t* map** |
| --- | --- |
| **Angry_vs_Neutral** | <https://neurovault.org/collections/RLWUZRQN/images/110709/> |
| **Happy_vs_Neutral** | <https://neurovault.org/collections/RLWUZRQN/images/110710/> |
| **Happy_vs_Baseline** | <https://neurovault.org/collections/RLWUZRQN/images/110711/> |
| **Angry_vs_Baseline** | <https://neurovault.org/collections/RLWUZRQN/images/110712/> |
| **Neutral_vs_Baseline** | <https://neurovault.org/collections/RLWUZRQN/images/110713/> |
| **All_vs_Baseline** | <https://neurovault.org/collections/RLWUZRQN/images/110714/> |

**Covariates to ROI effects, EMG responses and ratings**

We investigated the effect of the following covariates (self-ratings) to the main outcomes: Interpersonal reactivity index, empathic concern subscale (IRI-EC), perceived stress scale (PSS), Psychopathic Personality Inventory Revised, subscale cold-heartedness (PPI-R C),  Epworth Sleepiness Scale (ESS), Emotional Contagion scale (ECS) and Positive and Negative Affect Schedule (PANAS). The covariates were investigated across sleep conditions using mixed effects model where ROI effects, EMG responses or ratings were included as dependent variables (one at a time). Each model included individual subjects as random effects as well as fixed effects for block type, sleep condition and age group as well as the specific covariate of interest, i.e. the estimates represent bivariate associations between the covariate and dependent variable controlling for block type, sleep condition and age group.

**Supplementary table 5: Covariates to ROI effect estimates**

| **Amygdalae** | | | | | | | | | | |
| --- | --- | --- | --- | --- | --- | --- | --- | --- | --- | --- |
|  | **IRI EC** | | **PSS 14** | | **PPI-R C** | | **ESS** | | **ECS** | |
|  | **estimate_CI** | ***p*** | **estimate_CI** | ***p*** | **estimate_CI** | ***p*** | **estimate_CI** | ***p*** | **estimate_C** | ***p*** |
| **Happy_vs_Angry** | -0.149 [-0.917; 0.619] | 0.7 | -0.16 [-0.436; 0.116] | 0.25 | -0.254 [-0.861; 0.353] | 0.406 | -0.019 [-0.256; 0.217] | 0.872 | -0.18 [-1.184; 0.824] | 0.72 |
| **Happy_vs_Neutral** | 0.445 [-0.178; 1.068] | 0.159 | -0.001 [-0.228; 0.227] | 0.994 | -0.263 [-0.711; 0.185] | 0.245 | -0.008 [-0.202; 0.186] | 0.937 | 0.006 [-0.768; 0.78] | 0.987 |
| **Angry_vs_Neutral** | 0.593 [-0.046; 1.232] | 0.069 | 0.16 [-0.073; 0.392] | 0.175 | -0.009 [-0.523; 0.504] | 0.972 | 0.012 [-0.187; 0.21] | 0.906 | 0.186 [-0.701; 1.074] | 0.675 |
| **Happy_vs_Baseline** | 0.247 [-0.354; 0.848] | 0.415 | 0.253 [0.037; 0.47] | 0.023 | 0.258 [-0.2; 0.716] | 0.265 | 0.422 [0.238; 0.607] | 0 | -0.175 [-0.934; 0.585] | 0.646 |
| **Angry_vs_Baseline** | 0.396 [-0.196; 0.988] | 0.187 | 0.413 [0.2; 0.627] | 0 | 0.512 [0.032; 0.991] | 0.037 | 0.441 [0.26; 0.623] | 0 | 0.005 [-0.746; 0.757] | 0.989 |
| **Neutral_vs_Baseline** | -0.196 [-0.729; 0.337] | 0.465 | 0.254 [0.057; 0.451] | 0.012 | 0.521 [0.083; 0.959] | 0.02 | 0.429 [0.265; 0.594] | 0 | -0.181 [-0.879; 0.517] | 0.605 |
| **Happy_and_Angry_vs_Baseline** | 0.646 [-0.302; 1.593] | 0.178 | 0.666 [0.324; 1.008] | 0 | 0.77 [0.042; 1.497] | 0.038 | 0.863 [0.575; 1.151] | 0 | -0.169 [-1.299; 0.96] | 0.764 |

**Supplementary table 5 (continued)**

| **All_vs_Baseline** | 0.449 [-0.848; 1.746] | 0.492 | 0.92 [0.45; 1.391] | 0 | 1.291 [0.246; 2.336] | 0.016 | 1.292 [0.899; 1.685] | 0 | -0.35 [-1.93; 1.23] | 0.658 |
| --- | --- | --- | --- | --- | --- | --- | --- | --- | --- | --- |
| **Fusiform face area (left)** | | | | | | | | | | |
| **Happy_vs_Angry** | -0.251 [-0.977; 0.476] | 0.494 | -0.219 [-0.482; 0.043] | 0.1 | -0.367 [-0.925; 0.191] | 0.193 | -0.133 [-0.357; 0.091] | 0.24 | -0.156 [-1.093; 0.78] | 0.739 |
| **Happy_vs_Neutral** | 0.012 [-0.689; 0.714] | 0.972 | -0.127 [-0.377; 0.123] | 0.315 | -0.004 [-0.574; 0.565] | 0.988 | -0.011 [-0.226; 0.205] | 0.922 | 0.149 [-0.718; 1.016] | 0.731 |
| **Angry_vs_Neutral** | 0.263 [-0.328; 0.855] | 0.378 | 0.092 [-0.122; 0.306] | 0.392 | 0.363 [-0.109; 0.835] | 0.129 | 0.123 [-0.06; 0.305] | 0.184 | 0.306 [-0.488; 1.099] | 0.443 |
| **Happy_vs_Baseline** | 0.749 [-0.283; 1.78] | 0.152 | 0.651 [0.28; 1.023] | 0.001 | 0.799 [-0.064; 1.661] | 0.069 | 0.766 [0.448; 1.084] | 0 | 0.705 [-0.506; 1.916] | 0.248 |
| **Angry_vs_Baseline** | 0.999 [-0.007; 2.005] | 0.052 | 0.87 [0.506; 1.235] | 0 | 1.166 [0.324; 2.007] | 0.007 | 0.899 [0.589; 1.209] | 0 | 0.862 [-0.388; 2.111] | 0.172 |
| **Neutral_vs_Baseline** | 0.736 [-0.026; 1.499] | 0.058 | 0.778 [0.502; 1.054] | 0 | 0.803 [0.172; 1.433] | 0.013 | 0.777 [0.542; 1.012] | 0 | 0.556 [-0.396; 1.508] | 0.246 |
| **Happy_and_Angry_vs_Baseline** | 1.748 [-0.197; 3.693] | 0.077 | 1.521 [0.819; 2.224] | 0 | 1.964 [0.334; 3.595] | 0.019 | 1.665 [1.066; 2.265] | 0 | 1.567 [-0.777; 3.911] | 0.185 |
| **All_vs_Baseline** | 2.484 [-0.119; 5.086] | 0.061 | 2.299 [1.358; 3.241] | 0 | 2.767 [0.587; 4.948] | 0.014 | 2.442 [1.64; 3.244] | 0 | 2.123 [-1.017; 5.263] | 0.18 |

**Supplementary table 5 (continued)**

| **Fusiform face area (right)** | | | | | | | | | | |
| --- | --- | --- | --- | --- | --- | --- | --- | --- | --- | --- |
| **Happy_vs_Angry** | 0.199 [-0.527; 0.925] | 0.586 | -0.093 [-0.356; 0.17] | 0.482 | -0.506 [-1.069; 0.057] | 0.077 | -0.108 [-0.332; 0.116] | 0.341 | 0.107 [-0.776; 0.991] | 0.808 |
| **Happy_vs_Neutral** | 0.236 [-0.462; 0.933] | 0.502 | -0.025 [-0.278; 0.227] | 0.843 | -0.188 [-0.726; 0.35] | 0.487 | -0.058 [-0.271; 0.156] | 0.592 | 0.57 [-0.258; 1.397] | 0.173 |
| **Angry_vs_Neutral** | 0.037 [-0.566; 0.64] | 0.904 | 0.068 [-0.15; 0.286] | 0.536 | 0.318 [-0.159; 0.794] | 0.187 | 0.05 [-0.135; 0.235] | 0.592 | 0.462 [-0.257; 1.182] | 0.202 |
| **Happy_vs_Baseline** | 1.656 [0.654; 2.659] | 0.002 | 1.234 [0.869; 1.599] | <0.001 | 0.944 [0.142; 1.746] | 0.022 | 1.369 [1.06; 1.678] | 0 | 1.697 [0.584; 2.81] | 0.004 |
| **Angry_vs_Baseline** | 1.457 [0.384; 2.53] | 0.009 | 1.327 [0.939; 1.716] | <0.001 | 1.45 [0.565; 2.334] | 0.002 | 1.477 [1.149; 1.804] | 0 | 1.589 [0.345; 2.833] | 0.013 |
| **Neutral_vs_Baseline** | 1.421 [0.503; 2.339] | 0.003 | 1.259 [0.927; 1.592] | <0.001 | 1.132 [0.386; 1.878] | 0.004 | 1.427 [1.15; 1.703] | 0 | 1.127 [0.093; 2.161] | 0.033 |
| **Happy_and_Angry_vs_Baseline** | 3.114 [1.157; 5.07] | 0.002 | 2.562 [1.852; 3.271] | <0.001 | 2.393 [0.801; 3.985] | 0.004 | 2.845 [2.247; 3.444] | 0 | 3.286 [1.062; 5.51] | 0.005 |
| **All_vs_Baseline** | 4.534 [1.766; 7.302] | 0.002 | 3.821 [2.817; 4.825] | <0.001 | 3.525 [1.264; 5.787] | 0.003 | 4.272 [3.429; 5.115] | 0 | 4.413 [1.284; 7.542] | 0.007 |

**Supplementary table 6: Covariates to EMG responses**

| Muscle | Covariate | Estimate_CI | *p* |
| --- | --- | --- | --- |
| Corrugator | IRI_EC | -1.08e-04 [-3.10e-04, 9.40e-05] | 0.2897 |
| Corrugator | PPIR_C | 6.94e-06 [-1.22e-05, 2.61e-05] | 0.4711 |
| Corrugator | ESS | -1.66e-06 [-3.19e-05, 2.86e-05] | 0.9131 |
| Corrugator | PSS14 | -7.20e-06 [-2.27e-05, 8.32e-06] | 0.3571 |
| Corrugator | ECS | -3.90e-04 [-8.27e-04, 0.0000485] | 0.0799 |
| Corrugator | PANAS_Positive | -1.73e-06 [-1.95e-05, 1.60e-05] | 0.8466 |
| Corrugator | PANAS_Negative | -7.67e-06 [-2.58e-05, 1.05e-05] | 0.4022 |
| Zygomatic | IRI_EC | -4.73e-05 [-1.10e-04, 1.51e-05] | 0.1347 |
| Zygomatic | PPIR_C | 2.60e-06 [-3.44e-06, 8.63e-06] | 0.3931 |
| Zygomatic | ESS | 6.89e-07 [-8.52e-06, 9.89e-06] | 0.8816 |
| Zygomatic | PSS14 | 2.71e-06 [-2.01e-06, 7.43e-06] | 0.2560 |
| Zygomatic | ECS | -2.21e-05 [-5.07e-05, 6.47e-06] | 0.1262 |
| Zygomatic | PANAS_Positive | 1.12e-06 [-4.07e-06, 6.30e-06] | 0.6687 |
| Zygomatic | PANAS_Negative | 2.62e-06 [-3.02e-06, 8.27e-06] | 0.3566 |

**Supplementary table 7: Covariates to ratings**

| Type of rating | covariate | Estimate_CI | *p* |
| --- | --- | --- | --- |
| Happiness | IRI_EC | -2.343 [-7.975. 3.289] | 0.410 |
| Happiness | PPIR_C | 0.197 [-0.305. 0.698] | 0.437 |
| Happiness | ESS | -0.07 [-0.856. 0.716] | 0.859 |
| Happiness | PSS14 | -0.288 [-0.712. 0.137] | 0.181 |
| Happiness | ECS | -4.67 [-12.651. 3.31] | 0.245 |
| Happiness | PANAS_Positive | 0.129 [-0.353. 0.611] | 0.595 |
| Happiness | PANAS_Negative | -0.504 [-1.13. 0.123] | 0.113 |
| Angriness | IRI_EC | 5.763 [-0.245. 11.77] | 0.060 |
| Angriness | PPIR_C | -0.403 [-0.932. 0.127] | 0.134 |
| Angriness | ESS | -0.471 [-1.319. 0.378] | 0.272 |
| Angriness | PSS14 | 0.283 [-0.18. 0.745] | 0.227 |
| Angriness | ECS | 7.955 [-1.339. 17.25] | 0.092 |
| Angriness | PANAS_Positive | -0.056 [-0.581. 0.469] | 0.832 |
| Angriness | PANAS_Negative | 0.48 [-0.204. 1.164] | 0.166 |

**Habituation effects**

***Mimicry***

Habituation by order of block was observed as hypothesised for zygomatic responses (-0.0101 [-0.0177, -0.0026], *p* = 0.004, one-sided) as well as for corrugator responses (-0.0316 [-0.0681 0.0058], *p* = 0.04, one-sided).

***Habituation of ratings***


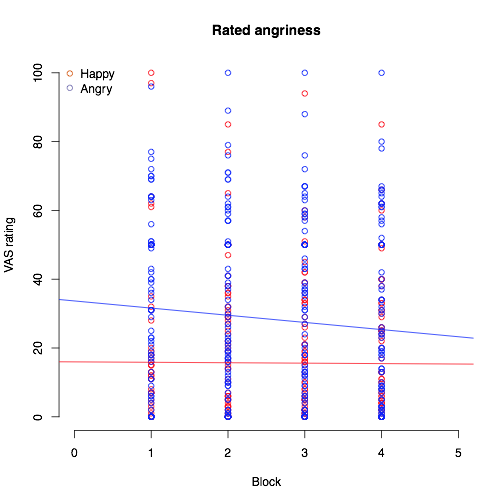

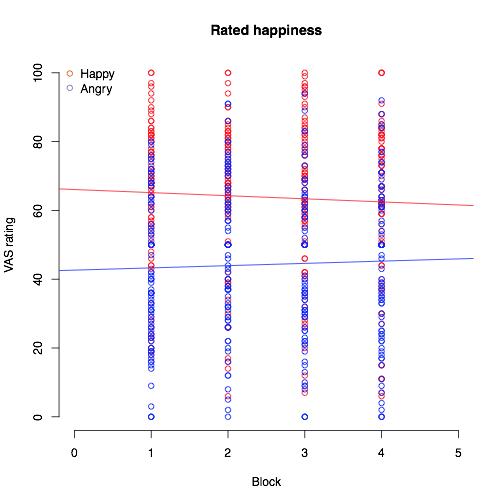
Ratings of angriness habituated over the course of the experiment (*p* = 0.048), but not ratings of happiness (*p* = 0.82

Supplementary figure 2. Habituation effects on ratings of happiness and anger

As suggested by one reviewer during the publication process, we performed analyses of the effect of partial sleep deprivation zygomatic responses and rated angriness on the first half of the experiment only, which did not notably affect the results presented in the main manuscript.

***Order effects***

As suggested by another reviewer, we also investigated whether the order (first or second scanning session) was significantly associated to the main behavioural outcomes. Although, the second session was significantly associated with lower ratings of both happiness and angriness, including session as a covariate in the final analyses didn’t noteworthily change the results. For EMG responses, session was not significantly associated with any of the responses.

***Rated emotional state***

Participants completed PANAS ratings at baseline and before scanning on each occasion. Sleep deprivation, compared to full sleep, caused a decrease in PANAS positive ratings (-3.5 [-5.2, -1.9], *p* = 0.00007), while the effect of sleep deprivation on PANAS negative ratings was -0.1 [-0.7, 0.6], *p* = 0.82.


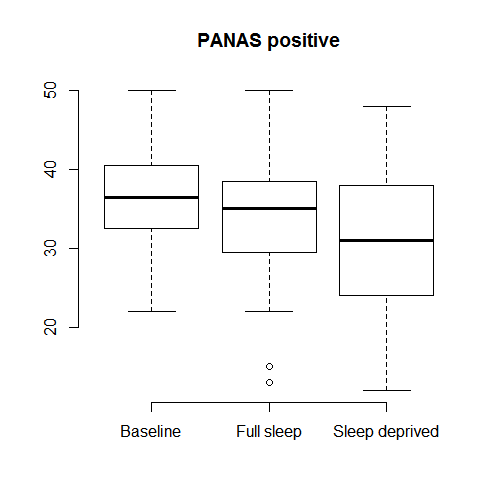

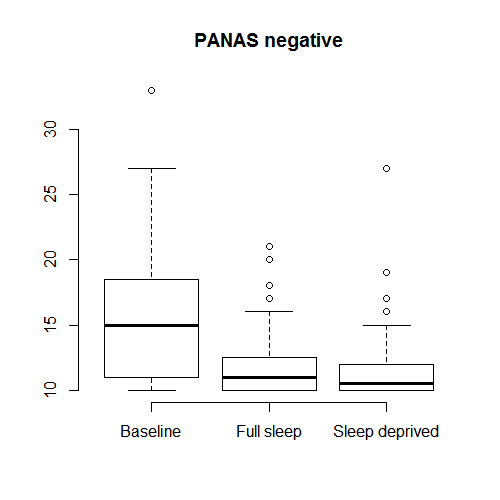


Supplementary figure 3. PANAS ratings

***Pupil diameter***

Eye-tracking was recorded from 138 sessions in 72 participants. To remove eyeblinks and episodes when the tracker lost the pupil, all records of pupil height and width where the first derivative was < -3 or > 3 were discarded, along with one consecutive data point before and after. Furthermore, all records of pupil height and width < 0.1 cm and > 0.3 cm were discarded as artefactual. Only sessions with > 50 % of the data existing after these cutoffs were used. If at least 50% of data remained in a window from 2 seconds before each block onset to 2 seconds after the end of the block, a loess curve was fitted to impute the missing data and down-sample the time-course. Pupil height and width were averaged to yield a pupil diameter measure. With 822 blocks from 70 sessions in 50 participants, a nested mixed-effects model was fitted.

The effect of happy faces vs neutral faces was 0.003 cm (0.000, 0.007), *p* = 0.048 and the effect of angry vs neutral faces was 0.004 cm (0.000, 0.007), *p* = 0.03201. The main effect of sleep restriction was not significant with a decrease of -0.004 cm (-0.009, 0.002), *p* = 0.181. Likewise, the sleep restriction*condition interaction was not significant.

**
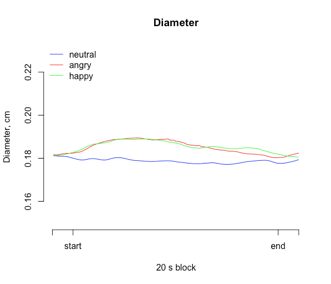
**

Supplementary figure 4. Pupil diameter

**Supplementary table 8. Pupil diameter, full model**

|  | Value | CI | *p*-value |
| --- | --- | --- | --- |
| (Intercept) | 0.181 | 0.173-0.189 | 0.000 |
| stimulus_angry | 0.004 | 0.000-0.007 | 0.032 |
| stimulus_happy | 0.003 | 0.000-0.007 | 0.048 |
| condition_Sleep_Deprived | -0.004 | -0.009-0.002 | 0.181 |
| Age_Group_Old | 0.009 | -0.007-0.025 | 0.262 |
| stimulus_angry:condition_Sleep_Deprived | 0.004 | -0.003-0.011 | 0.253 |
| stimulus_happy:condition_Sleep_Deprived | 0.004 | 0.004-0.011 | 0.212 |
| stimulus_angry:Age_Group_Old | -0.010 | -0.017--0.004 | 0.003 |
| stimulus_happy:Age_Group_Old | -0.011 | -0.017--0.004 | 0.003 |
| condition_Sleep_Deprived:Age_Group_Old | -0.002 | -0.013-0.009 | 0.687 |
| stimulus_angry:condition_Sleep_Deprived:Age_Group_Old | 0.003 | -0.011-0.017 | 0.663 |
| stimulus_happy:condition_Sleep_Deprived:Age_Group_Old | 0.007 | -0.007, 0.021 | 0.302 |

***Heart rate***

Heart rate was recorded from 158 sessions in 85 participants. Heart rate was determined based on recorded pulse events and was investigated within a time window of 20 s (each block). Time courses were inspected for each participant and recordings judged as excessively noisy were excluded. Heart rates < 40 beats per minute (bpm) or > 100 bpm were considered non-physiological and were censored. Recordings with less than 50% of data remaining were excluded at this stage. Where possible, censored data were imputed using carry-forward of the last non-censored heart rate. With 1655 blocks from 139 sessions in 76 participants, a nested mixed-effects model was fitted.

The effect of happy or angry vs neutral faces on heart rate was not significant. Neither the effect of sleep restriction or sleep*condition interaction was significant.

**
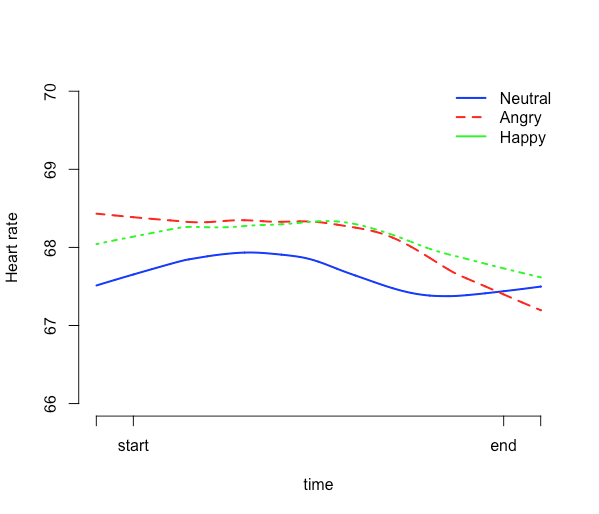
**

Supplementary figure 5. Heart rate

**Supplementary table 9. Heart rate, full model**

|  | Value | CI | *p*-value |
| --- | --- | --- | --- |
| (Intercept) | 67.731 | 64.77-70.70 | 0 |
| stimulus_Angry | -0.147 | -0.87-0.58 | 0.692 |
| stimulus_Happy | -0.043 | -0.77-0.68 | 0.907 |
| condition_Sleep_Deprived | -0.513 | -3.71-2.68 | 0.749 |
| Age_Group_Old | -0.824 | -5.24-3.59 | 0.711 |
| stimulus_Angry:condition_Sleep_Deprived | 0.406 | -0.61-1.42 | 0.431 |
| stimulusHappy:condition_Sleep_Deprived | 0.791 | -0.22-1.80 | 0.125 |
| stimulus_Angry:Age_Group_Old | 1.102 | 0.04-2.16 | 0.042 |
| stimulus_Happy:Age_Group_Old | 0.973 | -0.09-2.03 | 0.072 |
| condition_Sleep_Deprived:Age_Group_Old | 1.974 | -2.76-6.71 | 0.408 |
| stimulus_Angry:condition_Sleep_Deprived:Age_Group_Old | -0.634 | -2.14-0.87 | 0.409 |
| stimulus_Happy:condition_Sleep_Deprived:Age_Group_Old | -1.486 | -2.99-0.02 | 0.053 |
